# C2‐linked alkynyl poly‐ethylene glycol(PEG) adenosine conjugates as water‐soluble adenosine receptor agonists

**DOI:** 10.1111/cbdd.14128

**Published:** 2022-08-22

**Authors:** Lindsay Ferguson, Nasrin Shokrzadeh Madieh, Alexandra Vaideanu, Andreas Schatzlein, Joseph Festa, Harprit Singh, Geoffrey Wells, Sanjib Bhakta, Federico Brucoli

**Affiliations:** ^1^ School of Science University of the West of Scotland Paisley UK; ^2^ Leicester School of Pharmacy De Montfort University Leicester UK; ^3^ UCL School of Pharmacy University College London London UK; ^4^ Leicester School of Allied Health Sciences De Montfort University Leicester UK; ^5^ Department of Biological Sciences, Institute of Structural and Molecular Biology Birkbeck, University of London London UK

**Keywords:** adenosine receptor agonists, anti‐mycobacterial activity, NMR spectroscopy, poly‐ethylene glycol, purinergic receptor, Sonogashira cross‐coupling reaction

## Abstract

A series of 12 novel polyethylene‐glycol(PEG)‐alkynyl C2‐adenosine(ADN) conjugates were synthesized using a robust Sonogashira coupling protocol and characterized by NMR spectroscopy and mass spectrometry analysis. The ADN‐PEG conjugates showed null to moderate toxicity in murine macrophages and **12c** was active against *Mycobacterium aurum* growth (MIC = 62.5 mg/L). The conjugates were not active against *Mycobacterium bovis* BCG. Conjugates **10b** and **11b** exhibited high water solubility with solubility values of 1.22 and 1.18 mg/ml, respectively, in phosphate buffer solutions at pH 6.8. Further, **10b** and **11b** induced a significant increase in cAMP accumulation in RAW264.7 cells comparable with that induced by adenosine. Analogues **10c**, **11c** and **12c** were docked to the A_1_, A_2A_, A_2B_ and A_3_ adenosine receptors (ARs) using crystal‐structures and homology models. ADN‐PEG‐conjugates bearing chains with up to five ethyleneoxy units could be well accommodated within the binding sites of A_1_, A_2A_ and A_3_ ARs. Docking studies showed that compound **10b** and **11b** were the best A_2A_ receptor binders of the series, whereas **12c** was the best binder for A_1_ AR. In summary, introduction of hydrophilic PEG substituents at the C2 of adenine ring significantly improved water solubility and did not affect AR binding properties of the ADN‐PEG conjugates.

## Graphical abstract

Non‐cytotoxic C2‐linked alkynyl oligo ethylene glycol adenosine conjugates are highly water soluble and stimulate adenosine receptor activity.

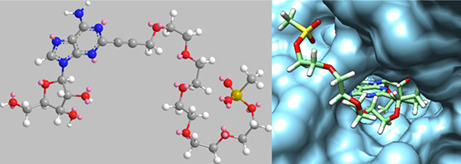

## 1 INTRODUCTION

Nucleosides are endogenous small molecules involved in key cellular processes and systems and are comprised of either purine (adenine and guanine) or pyrimidine (thymine, cytosine and uracil) nucleobases attached to a ribose moiety. Monophosphate‐nucleosides form the backbone of nucleic acid structures storing the genetic material of a whole organism, and monomeric nucleosides function as signalling molecules both inside the cell and in the extracellular matrix (Müller et al., 2020). Following ATP hydrolysis, adenosine (ADN) acts as a ligand for the adenosine receptors (ARs) family (Zimmermann, 2000). Adenosine receptors are G‐protein coupled receptors located on cellular outer membranes and are classed in four subtypes, A_1_, A_2A_, A_2B_ and A_3_ (Burnstock, 2007). Among the human ARs, A_1_ and A_3_ AR share 49% sequence similarity, whereas A_2A_ and A_2B_ AR share 59% similarity (Jacobson & Gao, 2006). Adenosine‐mediated activation of each ARs subtype leads to specific pharmacological effects, including regulation of vascular smooth muscle tone, blood flow, myocardial contractility and modulation of inflammatory responses, sleep and cognitive processes (Wu & Li, 2020; Reiss et al., 2019; El‐Tayeb et al., 2011; Antonioli et al., 2018; Urmaliya et al., 2009). Activation of A_1_ and A_2A_ ARs leads to inhibition of adenylyl cyclase activity, whereas activation of A_2A_ and A_2B_ stimulates the production of cAMP (Jacobson & Gao, 2006).

In the early 1990s, it was postulated that targeting AR might have potential therapeutic benefits in treating cardiovascular and inflammatory disorders. However, to date, only one synthetic, A_2A_ AR agonist, Regadenoson (**1**, CVT‐3146, Lexiscan), has been approved for human use as a radionuclide for myocardial perfusion imaging (Cabrera et al., 2013).

**FIGURE 1:**
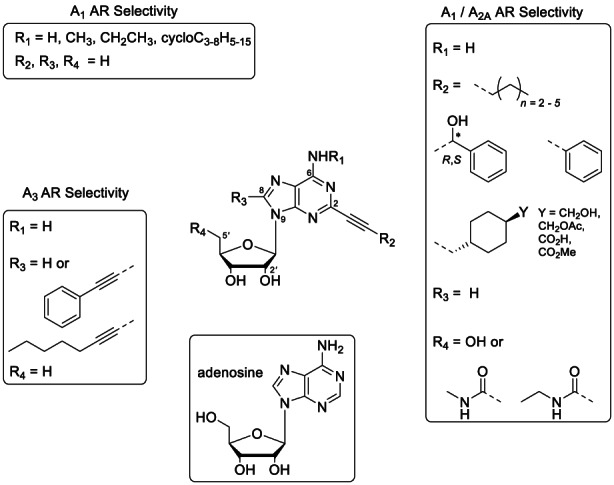
Structures of adenosine and selected AR agonists reported from the Matsuda, Cristalli and Macdonald groups from mid‐1990s to mid‐2000s. Alternating substitutions at C2‐, C8‐ and *N* ^6^‐position of the adenine ring and the presence of the urethane capping group at the 5′‐position of the ribose moiety influenced the selectivity of the adenosine derivatives leading to A_1_‐, A_2A_‐ and A_3_‐selective AR agonists.

**FIGURE 2:**
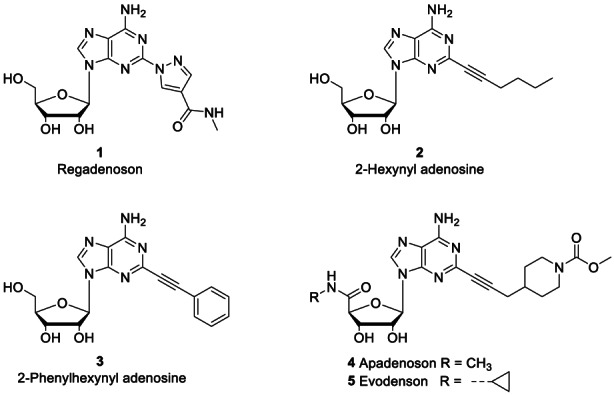
Pyrazole‐including ADN analogue regadenoson (**1**) is the only (moderately) A_2A_ AR selective agonist approved for medical use to date. Structures of A_1_/A_2A_ AR selective agonists incorporating alkynyl units at the C2‐position of the adenine ring, for example 2‐hexynyl and 2‐ phenylethynyl adenosine derivatives **2** and **3**, respectively, apadenoson (**4**) and evodenoson (**5**).

The design of ARs agonists has been mainly focussed on modifications of the ADN chemical scaffold. The introduction of substituents at the C2 and *N* ^6^‐positions of the adenine ring and at the 5′‐position of the ribose residue directed the selectivity of the parent compound (ADN) towards specific AR subtypes. Early investigations on structure activity relationships (SAR) of the adenine ring showed that introduction of alkynyl residues, containing either an aliphatic chain or aromatic residues, at the nucleobase C2 position resulted in analogues endowed with high affinity for A_1_ and A_2A_ ARs (Figure 1) (Volpini et al., 2002; Abiru et al., 1992; Cristalli et al., 1992). In particular, it was found that adenine C2‐bulky and hydrophobic substituents enhanced the A_2A_ AR‐selectivity of the ADN derivatives (Jacobson & Gao, 2006), which included hexynyl‐ (**2**) or phenylethynyl‐ (**3**) C2‐adenosine compounds, imaging contrast agent apadenoson (**4**) (Rieger et al., 2001; Zoghbi & Iskandrian, 2012) and multiple myeloma clinical candidate evodenoson (**5**) (Figure 2) (Rickles et al., 2010).

Incorporation of cycloalkyl substituents at the *N* ^6^‐position of adenosine led to analogues, for example sonedenoson, with an increased agonist activity toward A_1_ AR (Vittori et al., 2000; Mason & DiMarco, 2009). On the contrary, analogues bearing alkynyl chains at the C8 position of the adenine ring were found to exhibit selective A_3_ AR antagonistic activity (Volpini et al., 2001). Further to this, ADN derivatives containing both C2‐alkynyl and *N* ^6^‐methoxy or ‐methyl residues, or a single C2‐phenylethynyl chain (**3**) in the adenine ring with no *N* ^6^‐substitutions, showed increased affinity for A_3_ AR (Volpini et al., 2002; Volpini et al., 2007).

Recently, there has been a renewed interest in adenosine‐derived AR agonists, due to the roles played by ARs in neurogenerative diseases (e.g. Parkinson), neuroprotection, autoimmune inflammation, osteoarthritis and cancer (Jacobson et al., 2018; Congreve et al., 2018; Müller et al., 2018; Jacobson et al., 2019; Zheng et al., 2019). Mice deficient of A_2A_ AR were shown to develop osteoarthritis. ADN‐functionalized (hydrophilic) PEG‐nanoparticles were designed to prolong ADN half‐life and exhibited enhanced AR agonist activity, improving osteoarthritis conditions in rats (Liu et al., 2019). Activation of A_3_ AR has been correlated with tumour growth inhibition and apoptosis induction (Borea et al., 2015; Baraldi et al., 2012), and A_3_ AR agonists were found to display anti‐inflammatory and anticancer effects in melanoma, colon and prostate carcinoma disease models (Fishman et al., 2002; Valdés Zurita et al., 2018).

Previous AR ligands incorporating alkynyl hydrophobic units at the C2 position of the adenine residue, although exhibiting good AR selectivity, displayed poor water solubility (Rieger et al., 2001; Zoghbi & Iskandrian, 2012). Here, to address this shortcoming, we introduced PEG units in the adenosine core. The purpose of this work was to synthesize PEGylated adenosine analogues, measure their water‐solubility, investigate their ability to stimulate accumulation of cAMP in murine macrophages and evaluate their cytotoxicity and anti‐mycobacterial activity. Interactions of the ADN‐conjugates with A_1_, A_2A_, A_2B_ and A_3_ ARs binding pockets were studied using molecular modelling techniques.

Through molecular docking experiments, we sought to determine whether analogues containing hydrophilic, flexible alkynyl‐PEG arms might be still accommodated within ARs binding pockets and have selectivity towards a specific AR subtype, that is A_1_, A_2A_ or A_3_. The water‐soluble AR‐binding agents presented here can be employed in cellular‐based assays as tool‐compounds to further investigate the structures of adenosine receptors.

## 2 EXPERIMENTAL

See Supporting information for Data S1.

## 3 DISCUSSION

### 3.1 Chemistry

Polyethylene glycol (PEG) linkers are employed in the drug development process to improve the pharmaceutical properties of biotherapeutic agents due to their biostability and high water‐solubility. Bulky, water‐soluble PEG linkers were introduced at the adenine C2‐position of novel ADN‐alkynyl‐PEG derivatives.

**SCHEME 1:**
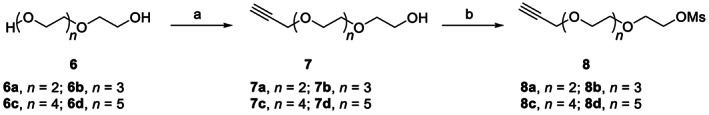
Synthetic approach towards the synthesis of the mesylated alkynyl PEG_3‐6_ units (**8a‐d**). *Reagents and conditions*: (a) propargyl bromide, NaH, THF, 0°C‐RT, 16 h; (b) MsCl, Et_3_N, DCM, 0°C‐RT, 4 h.

The synthesis of four polyethylene glycol linkers was conducted starting from commercially available ethylene glycol polymers containing three to six ethyleneoxy units (**6a‐d**). Polymers **6a‐d** were treated with propargyl bromide and a dispersion of sodium hydride in mineral oil. The resulting mono alkynyl‐PEG_3‐6_ **7a‐d**, which was purified by column chromatography and recovered in high yield, was converted to the tri‐, tetra‐, penta‐ and hexa‐ethylene glycol mesylated derivatives **8a‐d** (Scheme 1).

**SCHEME 2:**
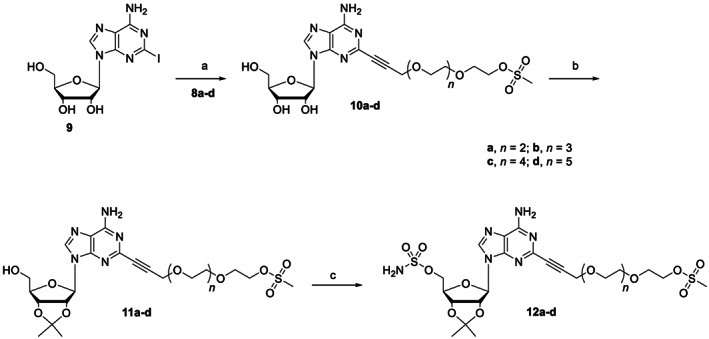
Synthesis of **12a‐d**. *Reagents and conditions*: (a) (PPh_3_)_2_PdCl_2_, CuI, Et_3_N, ACN (b) *p*‐TSA, dimethoxypropane, acetone; (c) NaH, H_2_NSO_2_Cl, dimethoxyethane.

Subsequently, the alkynyl‐PEG_3‐6_‐mesylated units **8a‐d** were installed at the C2 position of 2‐iodoadenosine **9**, which was synthesized as previously described (Ferguson et al., 2020) using established Sonogashira cross‐coupling reaction protocols to give derivatives **10a‐d**. The synthetic strategy proceeded with the protection of the C‐2′, C‐3′ and C‐5′ hydroxyl groups of the ribose ring in order to reduce the number of H‐Bond Donor (HBD) groups present in the compounds' frameworks. Protection of C‐2′, C‐3′ and C‐5′ OH‐groups in the **11a‐d** and **12a‐d** series was carried out to determine whether limited H‐bond interactions might affect the binding to amino acid residues within ARs binding sites. Acetonide protection of the ribose C‐2′‐ and C‐3′‐hydroxyl groups using 2,2′‐dimethoxypropane gave adenosine derivatives **11a‐d**, whereas sulfamoylation of the 5′‐hydroxyl residue afforded sulfamate end‐capped C2‐alkynyl‐PEG‐mesylate adenosine conjugates **12a‐d** (Scheme 2).

### 3.2 Solubility determination

The equilibrium solubility of ADN‐PEG‐conjugates was determined using a modified version of the shake‐flask method (Plöger et al., 2018). An excess of the ADN‐PEG‐conjugate was added to either phosphate buffer solution (PBS buffer) or deuterium‐depleted water (DDW) at a pH value of 6.8. The resulting suspension was shaken at 37°C until a (thermodynamic) equilibrium was reached between saturated solution and undissolved solid. Solubility quantification of the samples was achieved using high‐pressure liquid chromatography (HPLC) analysis. Within the linear concentration range (5.0–1000.0 μg/ml) used in our experiments, a good linearity value (*r* ^2^ > 0.9995) was achieved for the concentration plots of the ADN‐conjugates (see Supporting Information). The aqueous solubility of **10b** was found to be 1.33 mg/ml in DDW and 1.22 mg/ml in PBS, whereas **11b** solubility was 1.16 mg/ml in DDW and 1.18 mg/ml in PBS (Table 1).

**TABLE 1:** Aqueous solubility values of PEG‐AND conjugates **10b** and **11b** obtained using the shake‐flask method. Solubility quantification of the samples in either DDW or PBS was determined using HPLC analysis and results were calculated using 230 nm wavelength signal

Compound	Solubility (mg/ml) (mean ± SD) DDW	Solubility (mg/ml) (mean ± SD) PBS
**10b**	1.33 ± 0.32	1.22 ± 0.26
**11b**	1.16 ± 0.64	1.18 ± 0.19

High aqueous solubility can be a desirable characteristic in ADN‐based AR agonists, such as **10b** and **11b**. Polar, hydrophilic ADN‐derivatives might have better handling in cellular based or biochemical target assays, compared with hydrophobic analogues requiring initial dilutions in organic solvents, that is DMSO.

### 3.3 Stimulation of adenosine receptors in murine macrophages

**FIGURE 3:**
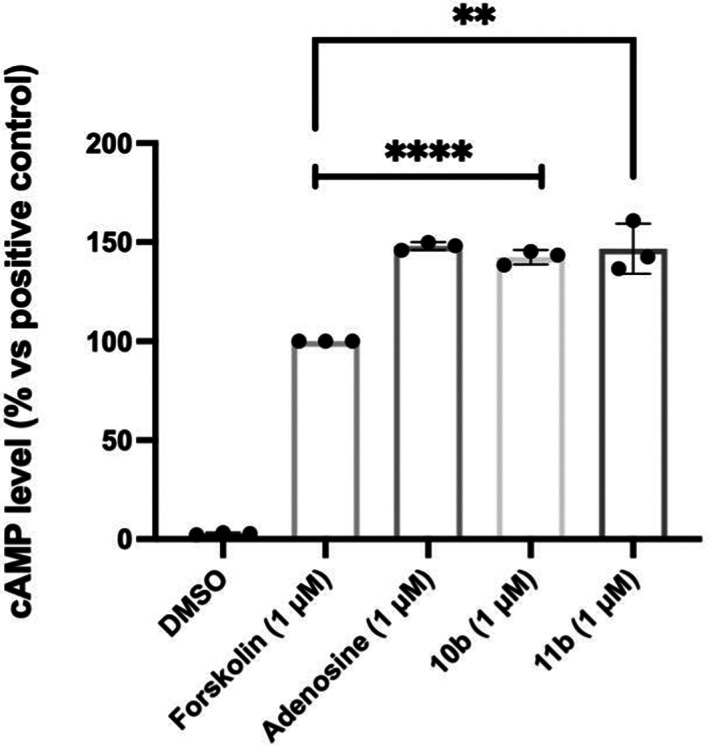
Adenosine, **10b** and **11b** increased cAMP production in RAW 261.7 murine macrophages. Macrophages were stimulated with either forskolin, adenosine, **10b** or **11b** for 10 min and cells were lysed. Levels of cAMP were measured from cell lysates using a cyclic AMP ELISA kit. Data are presented as mean and standard error of the mean (SEM) with cAMP levels shown as percentage change to that of positive control forskolin. Student's *t* test was performed with **** indicates significant cAMP change compared with positive control *p* < .0001 and ** indicates significant change *p* < .005.

Adenosine, **10b** and **11b** were tested for their ability to stimulate adenosine receptors by measuring cAMP accumulation in RAW264.7 cells after 10 minutes incubation. As illustrated in Figure 3, following incubation with RAW264.7 cells, **10b** and **11b** induced 42% (*p* < .0001) and 46% (*p* = .00331), respectively, increase in cAMP compared with forskolin. The ADN‐conjugates activity was found to be similar to that of adenosine, which induced a 48% (*p* < .0001) increase of cAMP levels compared with forskolin. However, it has to be noted that the cAMP production induced by **10b** and **11b** might be a result of A_2A_ or A_2B_ ARs stimulation and further studies should be conducted to ascertain whether the ligands might also activate A_1_ and A_3_ ARs, which conversely lead to cAMP inhibition.

### 3.4 NMR conformation studies

Nucleosides can adopt either *syn* or *anti* conformation, and purines can only be found as *syn* rotamers in the left‐handed, Z‐form of DNA structures (Sugiyama et al., 1996). In the *anti*‐conformer of adenosine, the H‐8 atom is positioned above the sugar ring, whereas in the *syn* conformer the N‐3 is found above the ribose moiety. Evaluation of *syn*/*anti* nucleoside conformers population can be carried out by analysing the chemical shifts (*δ*_H_) of the sugar ring H‐2′ (*δ*_H‐2′_) (Stolarski et al., 1980; Costanzi et al., 2003). Previous NMR spectroscopy studies showed that the H‐2*' δ*_H_ values of *anti*‐conformers ranged from 4.2 to 4.5 ppm, whereas in *syn* conformers *δ*_H‐2′_ ranged from 4.90 to 5.10 ppm (Stolarski et al., 1980; Costanzi et al., 2003). Although the *syn*/*anti* equilibrium is too rapid to be detected within the NMR‐timescale, *δ*_H‐2′_ can be determined as a mean value of the chemical shifts for the *syn*/*anti* conformations, for example the probability to find the nucleoside in one of the two rotamers.

**FIGURE 4:**
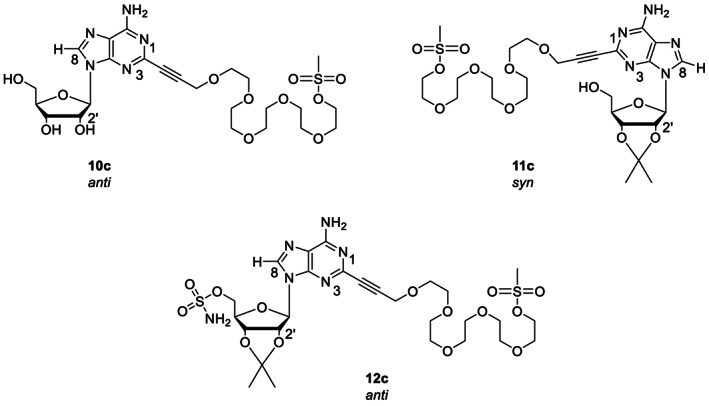
Molecular structures of PEG‐adenosine conjugates **10c**, **11c** and **12c** used in in silico molecular modelling and physicochemical properties prediction studies. Conjugate **10c** contained free ribose hydroxyl groups, whereas in **12c** all three ribose hydroxyl units were protected. In ADN derivative **11c** only the C‐2′‐ and C‐3′‐hydroxyl groups were protected.

Remarkably, derivative **12c**, despite bearing an acetonide protecting group, showed a *δ*_H‐2′_ value of 4.54 ppm, suggesting the adoption of an *anti*‐conformation (Figure 4). In this case, the steric hindrance caused by the sulfamoyl moiety attached to the C‐5′‐OH might impede the adenine ring to hover above the sugar unit (Table 2).

**TABLE 2:** Chemical shifts (*δ*_H_) of purine H‐8 and C6‐NH_2_ and ribose ring (H‐1′‐5′) protons of adenosine (ADN), 2‐iodo‐adenosine (**9**) and selected new ADN‐PEG conjugates **10a‐c**, **11b‐c** and **12c** ^a^

	C6‐NH_2_	H‐8	H‐1′	H‐2′	H‐3′	H‐4′	H‐5′
**ADN**		8.29	5.99	4.77	4.37	4.28	3.91
**9**	7.72	8.30	5.80	4.52	4.40	4.11	3.93
**10a**	7.49	8.42	5.86 (d)	4.53 (q)	4.32–4.30(m)	4.14–4.11 (m)	3.95 (q)
**10b**	7.50	8.43	5.86 (d)	4.53 (q)	4.31–4.29 (m)	4.13 (q)	3.95 (q)
**10c**	7.50	8.43	5.86 (d)	4.53 (q)	4.31–4.29 (m)	4.14–4.12 (m)	3.95 (q)
**11b**	7.52	8.42	6.09 (d)	4.94 (dd)	4.31–4.29 (m)	4.22 (td)	—
**11c**	7.51	8.41	6.09 (d)	4.94 (dd)	4.31–4.29 (m)	4.22 (td)	—
**12c**	—	8.29	6.24	4.54	4.36–4.34	4.13–4.11	3.81

^a^ Chemical shifts are reported and in ppm and were recorded on a JEOL 600 MHz in DMSO‐*d*_6_. Multiplicity is described as doublet (d), doublet of doublets (dd), triplet of doublets (td), triplet (t), quartet (q) and multiplet (m).

Interestingly, we have observed that the *δ*_H‐2′_ value for both **11b** and **11c** was 4.94 ppm, thus indicating that these adenosine derivatives might adopt a *syn* conformation in solution. This is probably due to the presence of the 2′,3′‐hydroxy acetonide protecting group that increases the attraction from the adenine ring N‐3 to the ribose H‐2′. This electronic pull resulted in a downfield shift of the H‐2′ proton signals of **11b** and **11c** (*δ*_H_ = 4.90 ppm), compared with **10a** and **10c** (*δ*_H_ = 4.53 ppm) that do not contain isopropylidene groups.

### 3.5 Cytotoxicity and anti‐mycobacterial activity

Nucleoside derivatives are extensively used in the clinic as antiviral and anticancer drugs, and several analogues containing modified nucleobase and sugar components were reported to exhibit promising antibacterial activity (Thomson & Lamont, 2019; Vitali et al., 2012). It was previously found that 2‐methyl adenosine inhibited the growth of *Mycobacterium tuberculosis* in a persistent state (Barrow et al., 2003), whilst 2‐iodo‐adenosine (**9**) exhibited bactericidal activity against *M. bovis* but not *M. aurum* (Ferguson et al., 2020). To this end, we sought to evaluate the anti‐mycobacterial activity of the novel ADN‐PEG conjugates against *M. aurum* and *M. bovis* strains using the HT‐SPOTi technique and isoniazid (INH) as a positive control (Gupta, 2012; Rizi et al., 2015; Danquah et al., 2016) (Table 3). Fast‐growing, non‐pathogenic *M. aurum* has an antibiotic‐susceptibility profile similar to *M. tuberculosis* and is routinely used as a rapid, convenient, high‐throughput surrogate model to test anti‐tubercular activity of new chemical agents (Gupta, 2012).

**TABLE 3:** Anti‐mycobacterial evaluation and cytotoxic activity evaluation of selected ADN‐PEG conjugates using the HT‐SPOTi technique

Compounds	GIC_50_ ^a^ RAW 264.7 (mg/L)	MIC_90_ ^b^ *M. aurum* (mg/L)	MIC_90_ *M. bovis* BCG (mg/L)^d^	SI^c^ GIC_50_/MIC_90_ *M. bovis* BCG
**9** ^d^	250	125	15	16
**10b**	250	250	250	1.0
**10c**	250	250	250	1.0
**11b**	62.5	500	500	0.1
**11c**	62.5	125	250	0.3
**12c**	31.2	62.5	500	0.1
**Isoniazid**	500	7.81	0.49	1020.4

^a^ GIC_50_ is the concentration of the compounds at which 50% of maximal inhibition of cell proliferation is achieved using resazurin‐based micro dilution method on murine macrophages RAW 264.7.

^b^ MIC_90_ is the lowest concentration of the compound at which 90% of the bacteria was inhibited.

^c^ SI is the ratio between GIC_50_ and the MIC_90 BCG_.

^d^ Data in agreement with previously published results (Ferguson et al., 2020).

The novel ADN‐PEG conjugates did not exhibit any notable antimycobacterial activity except from **12c** that was active against *M. aurum* with a MIC_90_ value of 62.5 mg/L. Conversely, 2‐iodo‐ADN (**9**), which was effective against *M. bovis* BCG (MIC_90_ = 15.6 mg/L) and had a selectivity index (SI) (e.g. the ratio between GIC_50_ and MIC_90_) of 16.

The conjugates were screened for cytotoxicity against RAW264.7 murine macrophages. Derivatives **10b** and **10c**, which had free ribose hydroxyl groups and contained PEG arms with four and five ethylene glycol units, respectively, were non‐cytotoxic against the mammalian cells (IC_50_ = 250 mg/L). ADN analogues **11b** and **11c**, which included the acetonide protecting unit at the C‐2′ ‐ C‐3′ diol, exhibited an IC_50_ value of 62.5 mg/L, whereas sulfamate‐end capped ADN analogue **12c** was found to be the most cytotoxic of the series with a IC_50_ value of 31.2 mg/L (Figure 4).

### 3.6 Docking experiments

Molecular modelling studies were conducted to ascertain whether the PEG units and protection of sugar OH‐groups might direct the selectivity of the ADN‐PEG conjugates towards a specific AR subtype. Previously reported ligands **2** and **3**, and novel analogues **10b**‐**c**, **11b**‐**c** and **12c** were docked to the A_1_, A_2A_, A_2B_ and A_3_ receptors using crystal structure‐derived coordinates for the A_1_ and A_2A_ receptors (PDB Ref 7LD4 and 2YDO, respectively) (Draper‐Joyce et al., 2021; Lebon et al., 2011) and the Alphafold (Jumper et al., 2021) structures for the A_2B_ and A_3_ receptors (https://www.uniprot.org) using Autodock Vina (Eberhardt et al., 2021; Trott & Olson, 2010; Alhossary et al., 2015). The A_2B_ and A_3_ receptors were energy minimized complexed with adenosine using AMBER (Case et al., 2022) prior to the docking calculations. The proteins were overlaid, and a binding site was defined using the adenosine bound in the 7LD4 and 2YDO structures as a reference. To test the docking parameters adenosine was redocked into the A_1_ and A_2A_ protein structures and gave an excellent overlay with the experimental structures (Figure S1). This docked orientation of adenosine was also reproduced for the A_2B_ and A_3_ proteins. Overall, the calculated binding affinities (Table 4) showed that the ligands were predicted to bind better to the A_2A_ receptor with the exception of **12c**, which had a preference for the A_1_ receptor. The two alkynyladenosine derivatives **2** and **3** were predicted to bind better overall and has the best docking scores for the A_2A_ receptor, consistent with their reported specificities (Volpini et al., 2002; Abiru et al., 1992; Cristalli et al., 1992).

**TABLE 4:** Calculated binding energies of ligands **2**, **3**, **10b**‐**c**, **11b**‐**c** and **12c** to the A_1_, A_2A_ and A_3_ adenosine receptors

Compound	Calculated binding energy (Kcal/mol)			
	A_1_ receptor	A_2A_ receptor	A_2B_ receptor	A_3_ receptor
**Adenosine**	−6.57	−7.39	−6.55	−6.53
**2**	−7.43	−8.42	−8.15	−7.64
**3**	−7.95	−8.52	−8.35	−8.26
**10b**	−6.54	−7.20	−7.18	−5.91
**10c**	−6.36	−7.00	−6.74	−6.45
**11b**	−7.01	−7.21	−7.35	−6.66
**11c**	−5.93	−7.00	−6.56	−6.19
**12c**	−6.01	−5.67	−6.03	−5.93

Ligands **10b**‐**c**, **11b**‐**c** and **12c** had higher binding energies (5.91–6.66 Kcal/mol) to A_3_ AR, and thus showed lower binding affinity, compared to alkynyladenosine derivatives **2** and **3**. Derivative **3** was predicted to be the better binder to A_3_ AR amongst the docked compounds. This confirms early findings showing that ADN analogues with C‐2phenylethynyl chain in the adenosine ring and without *N* ^6^‐substitutions had increased affinity for A_3_ AR (Volpini et al., 2007). Of the newly synthesized compounds, **11b** had the lowest docking scores for the four adenosine receptor subtypes and compound **10b** also had a low score for the A_2A_ and A_2B_ receptors. For compound **11b**, the PEG substituent was predicted to project from the adenosine binding site and sit on the solvent exposed surface of the protein (Figure S1). The purine stacks with a conserved phenylalanine residue in each of the binding pockets and the ribose sugar forms hydrogen bond interactions with adjacent residues. In contrast, **10b** has protected 2′ and 3′ hydroxyl groups and was predicted to adopt an alternative conformation in which the PEG linker occupies the usual adenosine binding site (Figure S1).

## 4 CONCLUSIONS

Adenosine receptors are involved in important cellular processes and adenosine‐derived AR agonists can serve as a platform for the development of therapeutic agents to treat neurogenerative diseases (e.g. Parkinson), cancer, autoimmune inflammation and osteoarthritis. Previous C2‐alkynyl substituted adenosine derivatives, for example **2**‐**5**, endowed with A_1_ / A_2A_ AR selectivity, suffered from low water solubility issues, with **2** and **3** exhibiting suboptimal distribution coefficient values.

High water solubility is an attractive feature in bio‐active molecules, and polyethyleneglycol residues might improve the hydrophilicity and distribution coefficient of AR agonists without affecting their AR binding properties and cytotoxicity.

Here, we used a robust Sonogashira cross‐coupling method to install PEG_3‐6_ arms at the adenine C2 site to furnish 12 novel ADN‐alkynyl‐PEG derivatives. The conjugates were generally non‐cytotoxic against murine macrophages. ADN‐PEG conjugates **10b** and **11b** were highly water soluble and stimulated a considerable increase in cAMP accumulation in RAW264.7 cells.

Molecular modelling studies revealed that **10b** and**11b** were the best A_2A_ receptor binder of the series, with **11b** having its PEG arm sitting on the solvent‐exposed surface of the protein. Ligand **12c** was predicted to have higher selectivity for the A_1_ receptor, although it had a lower predicted affinity than **11b**. In conclusion, **10b** was found to be best performing compound of the series, as it was non‐cytotoxic (IC_50_ = 250 mg/mL) and induced a 42% cAMP increase in RAW264.7 cells, had a water solubility value of 1.33 mg/ml and exhibited one of the lowest binding energy scores (−7.20 Kcal/mol) of the series for the A_2A_ receptor.

To the best of our knowledge, this is the first report of highly water‐soluble AR‐binding agents containing small pegylated units attached at the adenosine C2. Oligoethylene glycol substituents linked to adenosine might prolong the half‐life and increase agonistic activity of PEG‐conjugated purine ligands, which can be further tested for biophysical interactions with receptor targets and find applications as tool‐compounds to map structures of adenosine receptors.

## Supporting information


Supinfo

Data S1

Figure S1

## Data Availability

The data that supports the findings of this study are available in the supplementary material of this article
