# Supplementary material for: C2‐linked alkynyl poly‐ethylene glycol(PEG) adenosine conjugates as water‐soluble adenosine receptor agonists
Source: Chem Biol Drug Des. 2022 Aug 22;101(2):340–9. doi: 10.1111/cbdd.14128 (PMC10087458; doi:10.1111/cbdd.14128)
Supplement: Supplementary file 1 — Supinfo [file CBDD-101-340-s002.docx]

**Supporting Information**

**C2-linked alkynyl poly-ethylene glycol (PEG)adenosine conjugates as water-soluble adenosine receptor agonists**

Lindsay Ferguson, Nasrin Shokrzadeh Madieh, Alexandra Vaideanu, Andreas Schatzlein, Joseph Festa, Harprit Singh, Geoff Wells, Sanjib Bhakta and Federico Brucoli

**Table of contents**

Synthesis of mesylated alkynyl PEG_3-6_ units **8a-d**……………………………………….page 2

^1^H and ^13^C NMR spectra for selected compounds..………………………..…………….page 5

Protein alignment for docking experiments.………………...………………….………page 20

Plots for water solubility measurements……………..…………………………………page 24

**Synthesis of mesylated alkynyl PEG_3-6_ units 8a-d**

Synthesis of alkyne linkers

General procedure for the preparation of **7a-d**. To a solution of desired PEG **6a-d** (1 equiv.) in THF was added NaH (0.65 equiv.) and the mixture was stirred at room temperature for one hour. Propargyl bromide (1 equiv.) was added dropwise at 0 °C for 2 hours and stirred overnight at room temperature. The reaction was quenched with ice cold water and the mixture was then extracted with DCM (3 × 20 mL) and washed with brine (3 × 15 mL), dried over MgSO_4_, filtered and the solvent was removed *in vacuo*. Compounds **7a-d** were obtained as oils after purification by flash chromatography (2 to 10% MeOH in EtOH).

 **2-(2-(2-(prop-2-yn-1-yloxy)ethoxy)ethoxy)ethan-1-ol** **(7a).** Starting from 2.7 mmol of **6a**, 0.95 mmol of **7a** (180 mg, 35%) were recovered. A pale amber oil. *R_f_* = 0.37 (EtOH:MeOH / 9:1 *v/v*); MS m/z 189 (M^+^); ^1^H-NMR (400 MHz, CDCl_3_) *δ*_H_ 4.195 (d, *J* = 2.46 Hz, 2H), 3.71-3.64 (m, 10H), 3.61-3.59 (m, 2H), 2.43 ppm (t, *J* = 2.47 Hz, 1H).

 **3,6,9,12,15-pentaoxaoctadec-17-yn-1-ol (7c).**

Starting from 1.17 mmol of **6c**, 0.47 mmol of **7c** (130 mg, 40%) were recovered. A pale amber oil. MS m/z 277 (M^+^1); ^1^H-NMR (400 MHz, CDCl_3_) *δ*_H_ 4.19 (d, *J* = 2.52 Hz, 2H), 3.71-3.65 (m, 18H), 3.61-3.59 (m, 2H), 2.42 (t, *J* = 2.35 Hz, 1H).

 **3,6,9,12,15,18-hexaoxahenicos-20-yn-1-ol (7d).** Starting from 2.48 mmol of **6d**, 0.87 mmol of **7d** (280 mg, 35%) were recovered. A pale amber. *R_f_* = 0.11 (EtOH:MeOH / 9.8:0.2 *v/v*); MS m/z 321 (M^+^); ^1^H-NMR (400 MHz, CDCl_3_) *δ*_H_ 4.19 (d, *J* = 2.44 Hz, 2H), 3.72-3.64 (m, 23H), 3.61-3.58 (m, 2H), 2.42 (t, *J* = 2.40Hz, 1H).

General procedure for the preparation of **8a-d.**

To a solution of alkyne polyethylene glycol **7a-d** (1 equiv.) in CH_2_Cl_2_ (10 mL), a catalytic quantity of 4-dimethylaminopyridine (DMAP) was added under nitrogen atmosphere. Subsequently, Et_3_N (2 equiv.) and a solution of methanesulphonyl chloride (MsCl) (1.5 equiv.) in CH_2_Cl_2_ (5 mL) were added dropwise over 30 mins at 0 °C. After the addition was complete, the reaction mixture was stirred at 0 °C for 90 mins, then at room temperature for 4 h. The solvent of the reaction mixture was evaporated to dryness. The residue was redissolved in CH_2_Cl_2_ (25 mL) and washed with 3% HCl (25 mL) and brine (25 mL). The organic layer was separated, dried over MgSO_4_ and then concentrated *in vacuo*. Final compounds **8a-d** were purified by flash chromatography (0 to 10% MeOH in EtOAc).

 **2-(2-(2-(prop-2-yn-1-yloxy)ethoxy) ethoxy) ethylmethanesulfonate (8a).** Starting from alkyne triethylene glycol **7a** (0.80 mmol, 150 mg), **8a** (0.64 mmol, 170 mg, 80%) was recovered as a colourless oil. MS m/z 267 (M^+^1); ^1^H-NMR (400 MHz, CDCl_3_) *δ*_H_ = 4.39-4.37 (m, 2H), 4.20 (d, *J* = 2.36 Hz, 2H), 3.78-3.76 (m, 2H), 3.70-3.64 (m, 8H), 3.08 (s, 3H), 2.43 ppm (t, *J* = 2.32 Hz, 1H).

 **3,6,9,12-tetraoxapentadec-14-yn-1-yl methanesulfonate (8b).** Starting from alkyne tetraethylene glycol **7b** (0.89 mmol, 210 mg), **8b** (0.37 mmol, 116 mg, 42%) was recovered as a colourless oil. MS m/z 311 (M^+^1); ^1^H-NMR (400 MHz, CDCl_3_) *δ*_H_ = 4.38-4.36 (m, 2H), 4.19 (d, *J* = 2.47 Hz, 2H), 3.77-3.75 (m, 2H), 3.70-3.64 (m, 12H), 3.08 (s, 3H), 2.43 ppm (t, *J* = 2.40 Hz,1H).

 **3,6,9,12,15-pentaoxaoctadec-17-yn-1-yl methanesulfonate (8c).** Starting from alkyne pentaethylene glycol **7c** (1.08 mmol, 300 mg), **8c** (0.92 mmol, 328 mg, 85%) was recovered as a colourless oil. MS m/z 355 (M^+^1); ^1^H-NMR (600 MHz, CDCl_3_) *δ*_H_ = 4.36-4.35 (m, 2H), 4.18 (d, J = 2.4 Hz, 1H), 3.75-3.73 (m, 2H), 3.66-3.68 (2H), 3.64-3.66 (4H), 3.62-3.63 (4H), 3.61-3.62 (4H), 3.60-3.61 (2H), 3.06 (s, 3H), 2.42 (t, J = 2.4 Hz, 1H).

 **3,6,9,12,15,18-hexaoxahenicos-20-yn-1-yl methanesulfonate (8d).** Starting from alkyne hexaethylene glycol **7d** (1.05 mmol, 337 mg), **8d** (0.51 mmol, 205 mg, 49%) was recovered as a colourless oil. *R_f_* = 0.33 (EtOH:MeOH / 9.8:0.2 *v/v*); MS m/z 399 (M^+^1); ^1^H-NMR (400 MHz, CDCl_3_) *δ*_H_ = 4.39-4.36 (m, 2H), 4.20 (d, *J* = 2.32 Hz, 2H), 3.78-3.75 (m, 2H), 3.70-3.62 (m, 20H), 3.08 (s, 3H), 2.43 ppm (t, *J* = 2.55 Hz,1H).

**^1^H and ^13^C NMR spectra for selected compounds**

**(8c). ^1^H NMR spectrum**

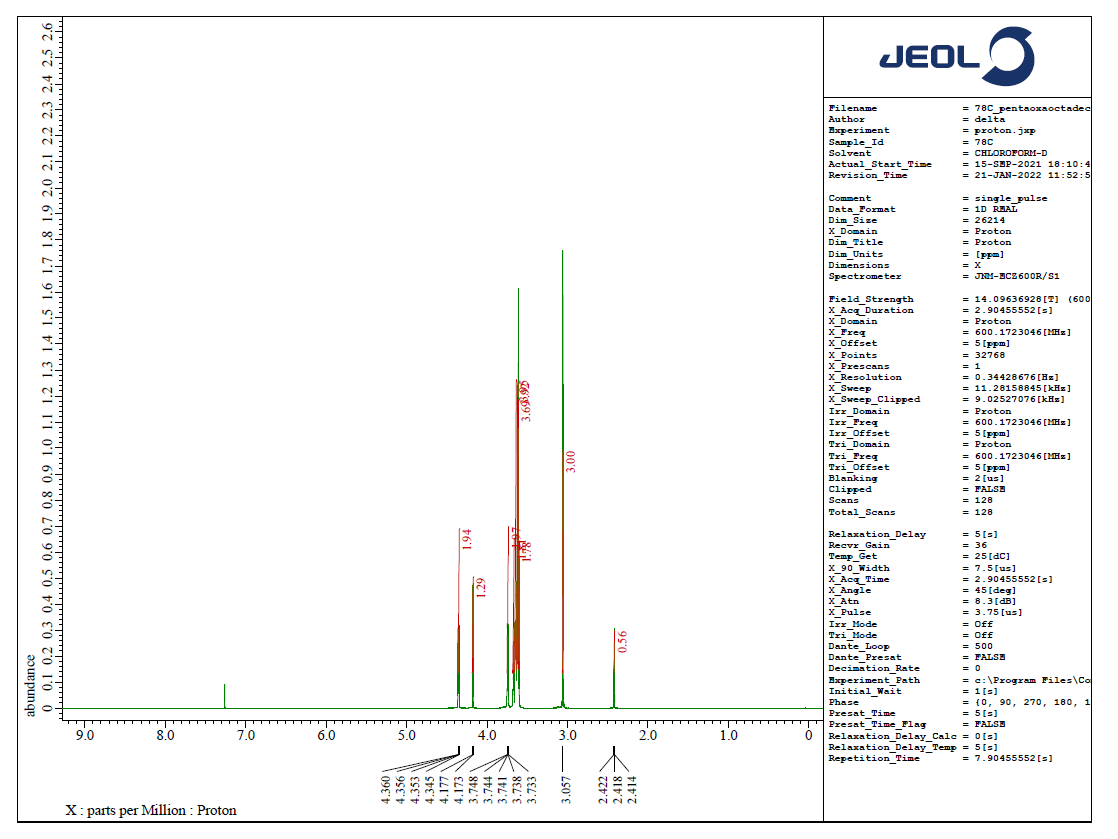


**10a. ^1^H NMR spectrum**

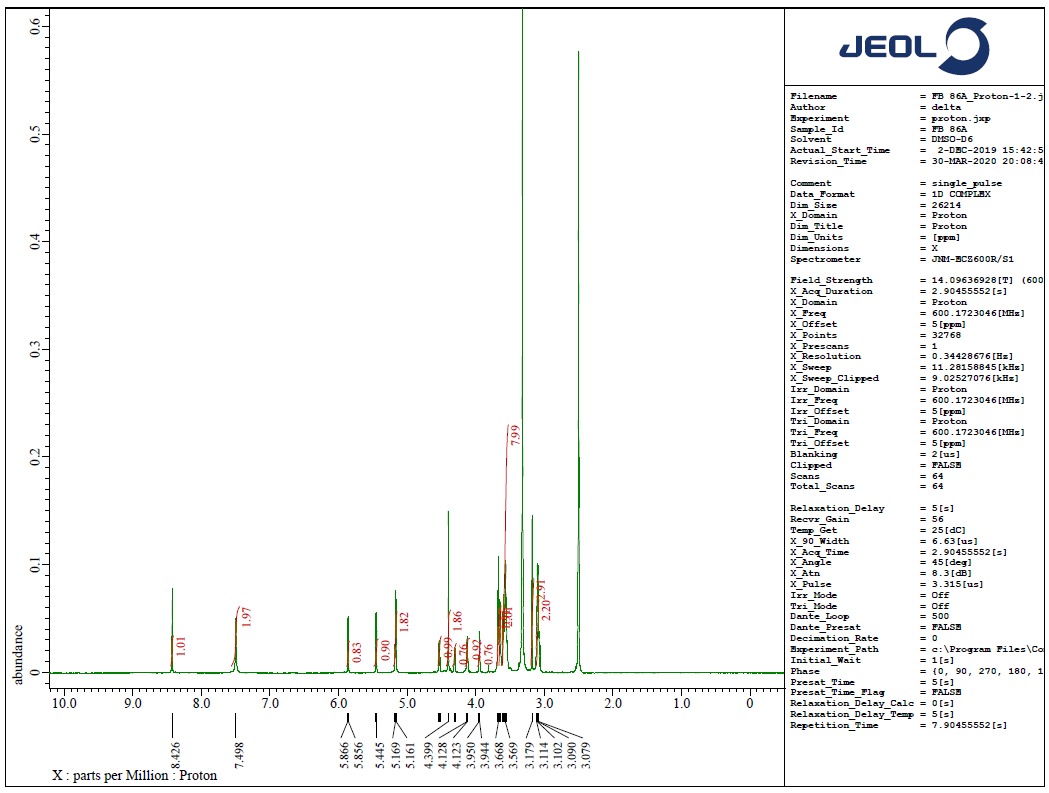


**10a. ^13^C NMR spectrum**


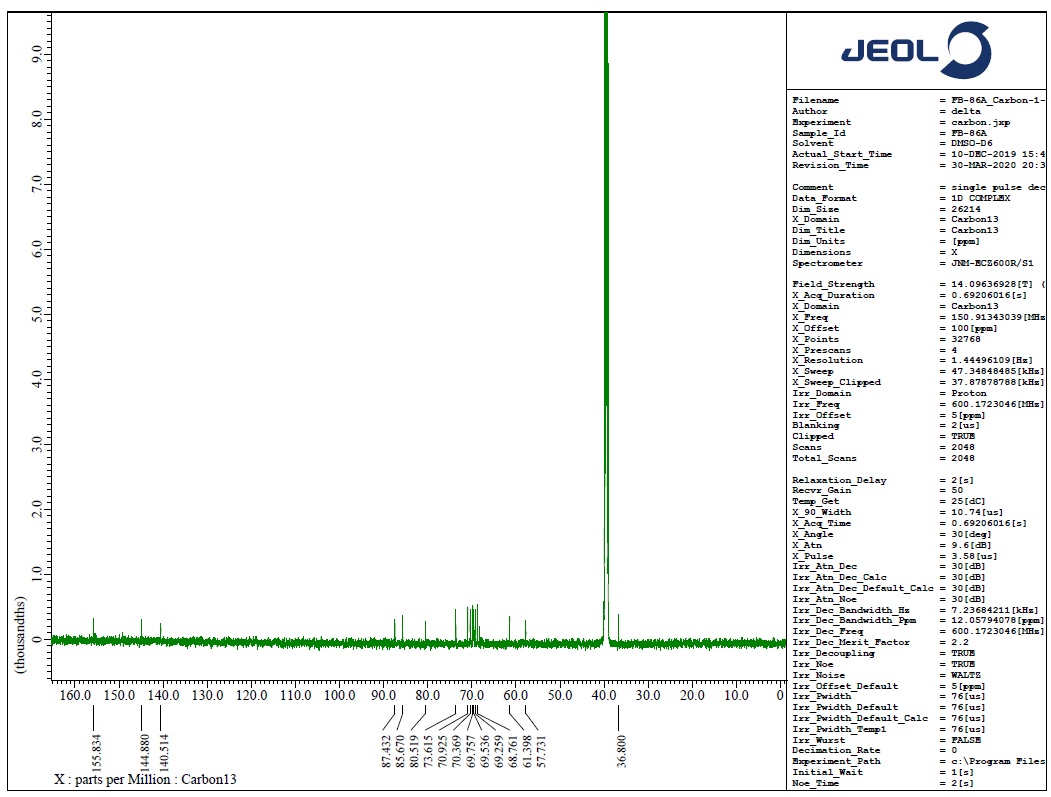


**10b. ^1^H NMR spectrum**

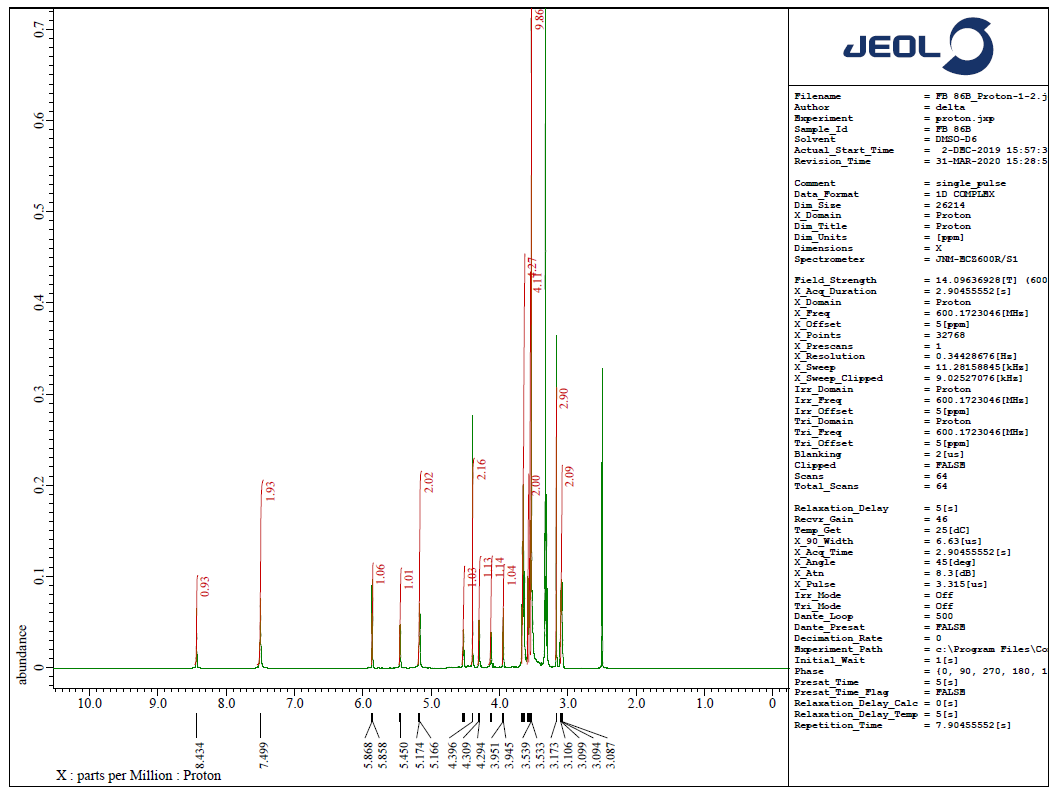


**10b. ^13^C NMR spectrum**


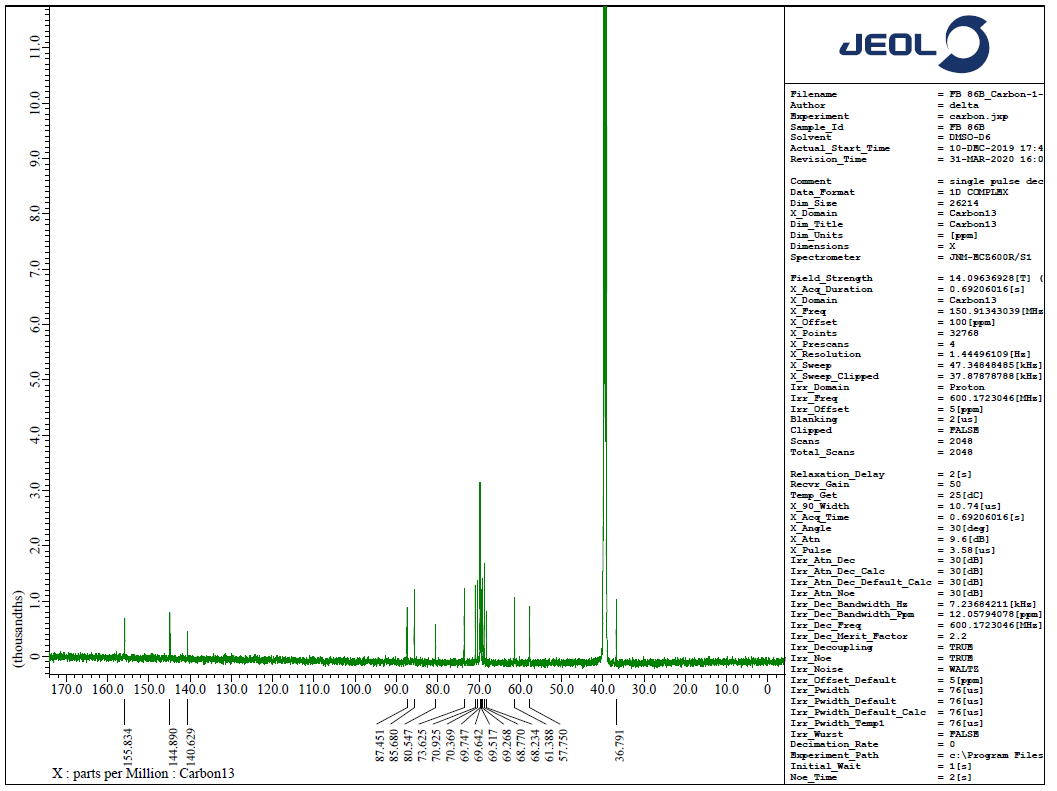


**10c. ^1^H NMR spectrum**

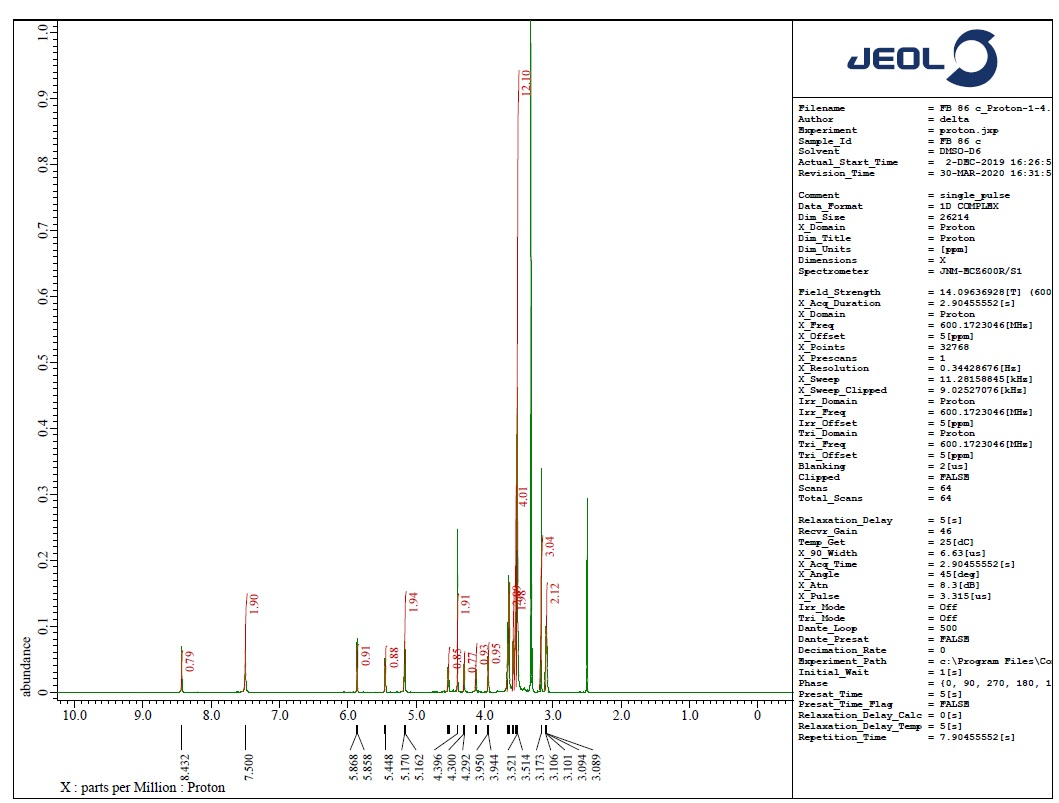


**10c. ^13^C NMR spectrum**


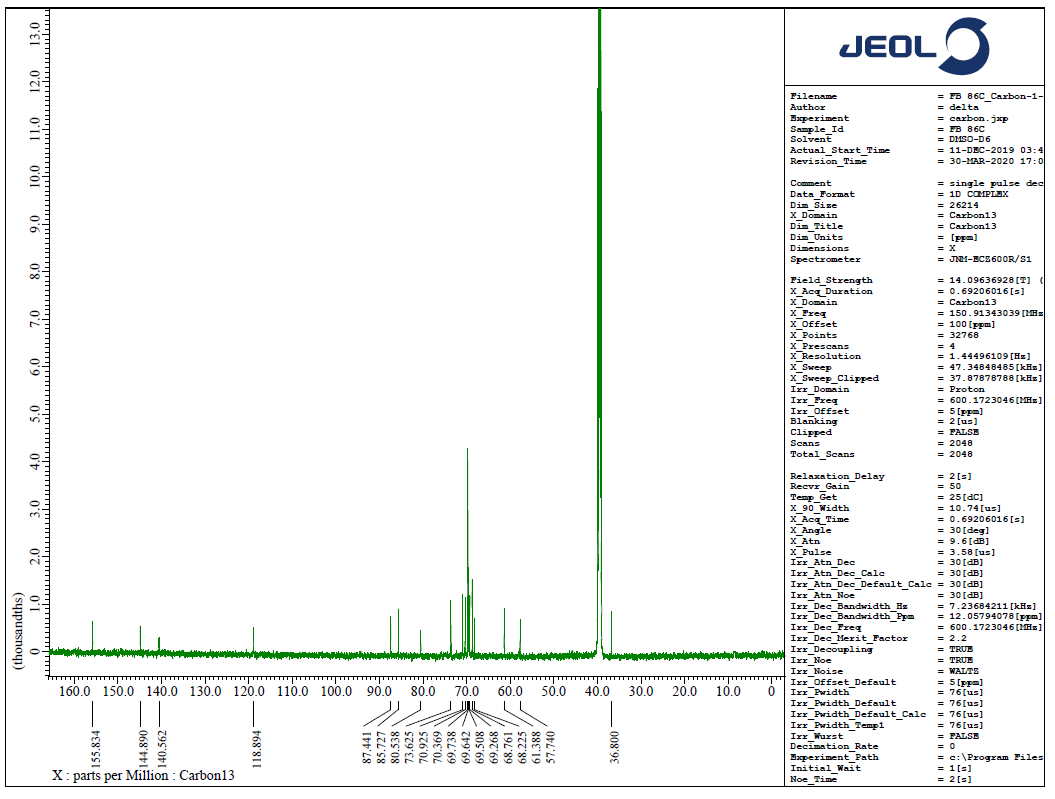


**10d.** **^1^H NMR spectrum**

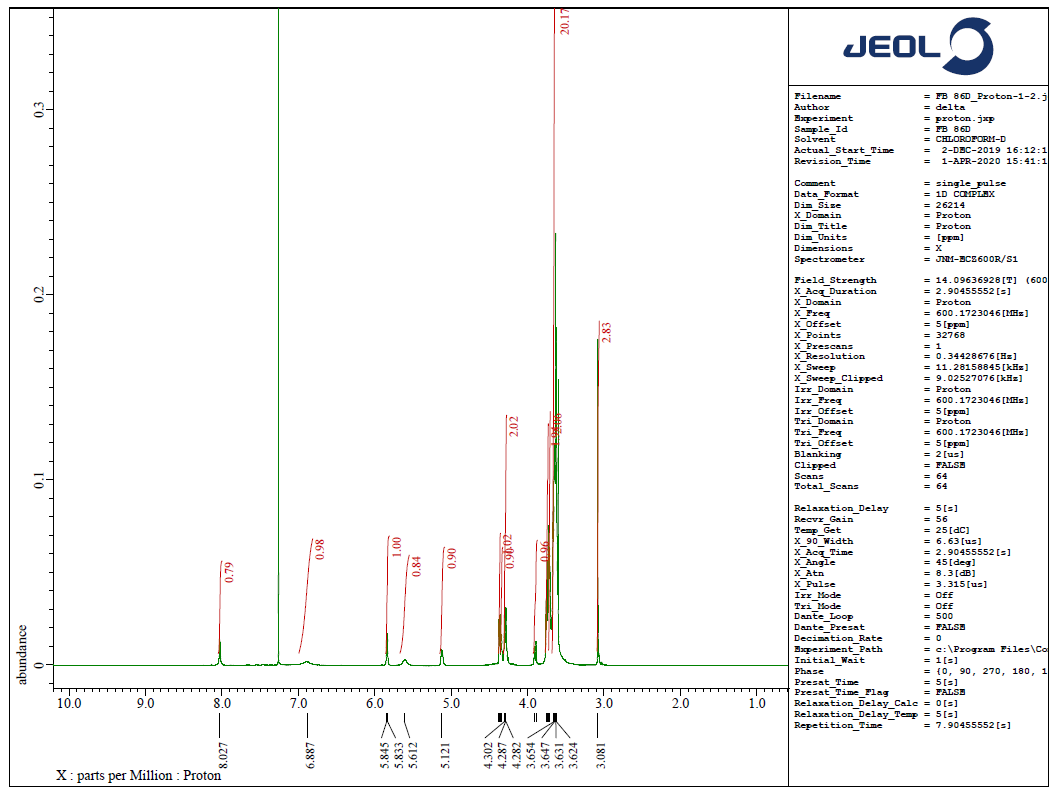


**10d. ^13^C NMR spectrum**


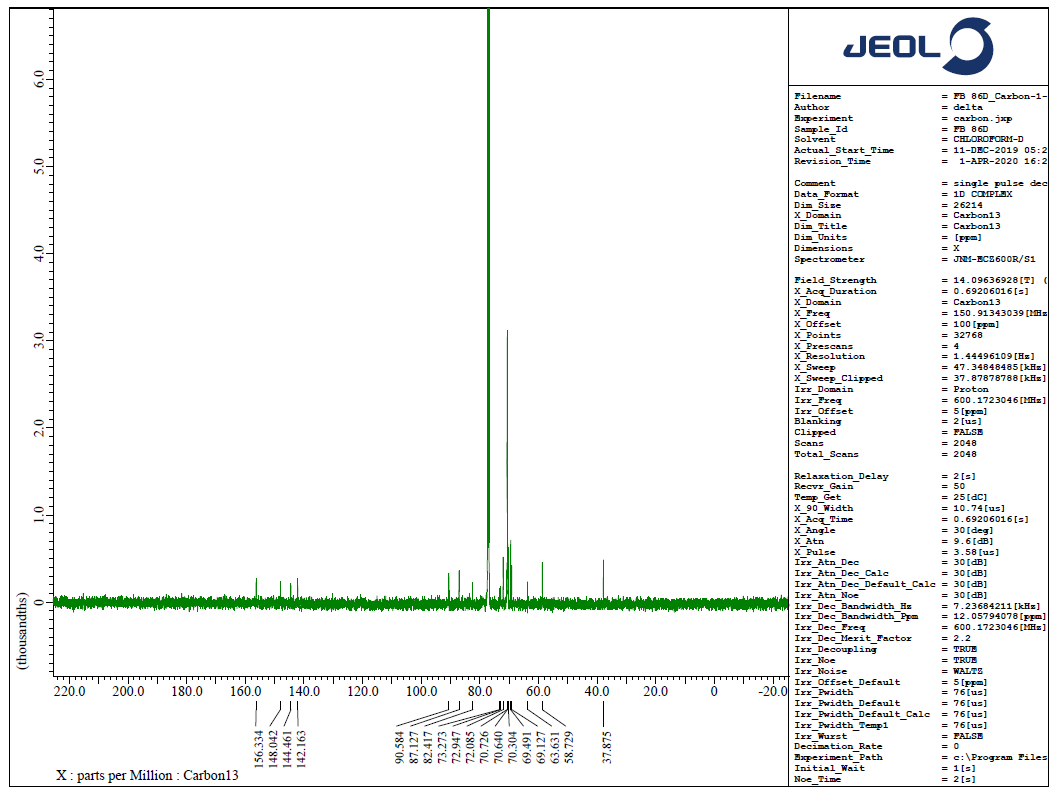


**11b. ^1^H NMR spectrum**

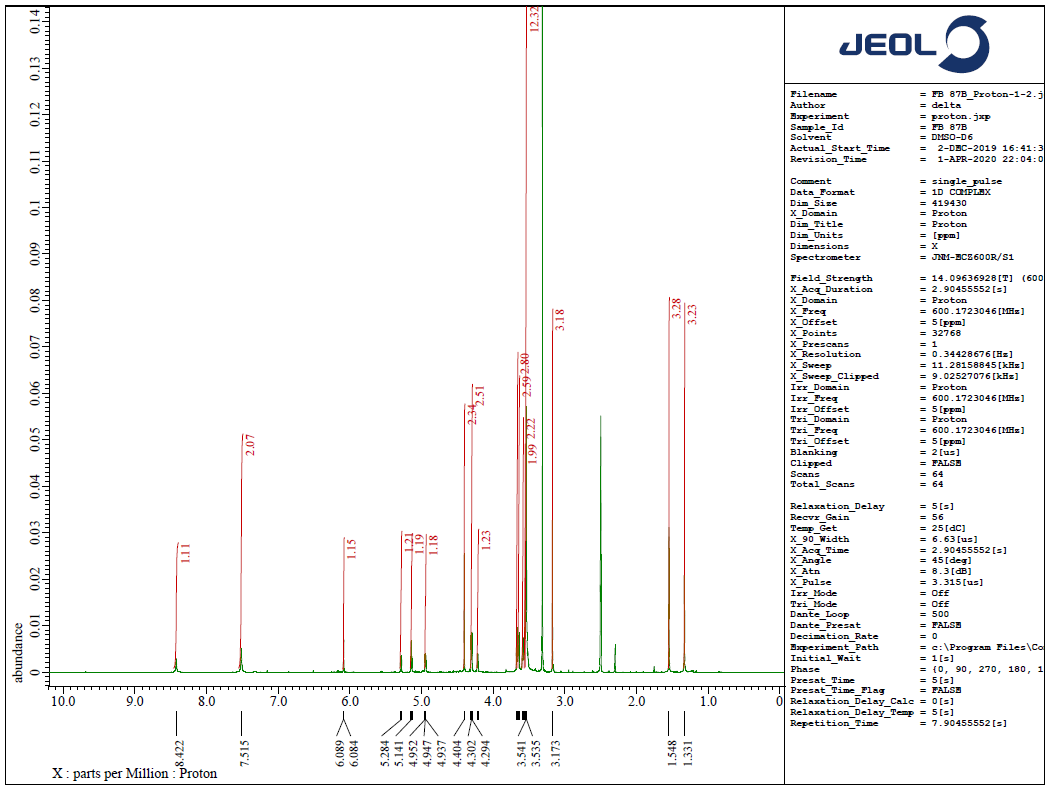


**11b. ^13^C NMR spectrum**


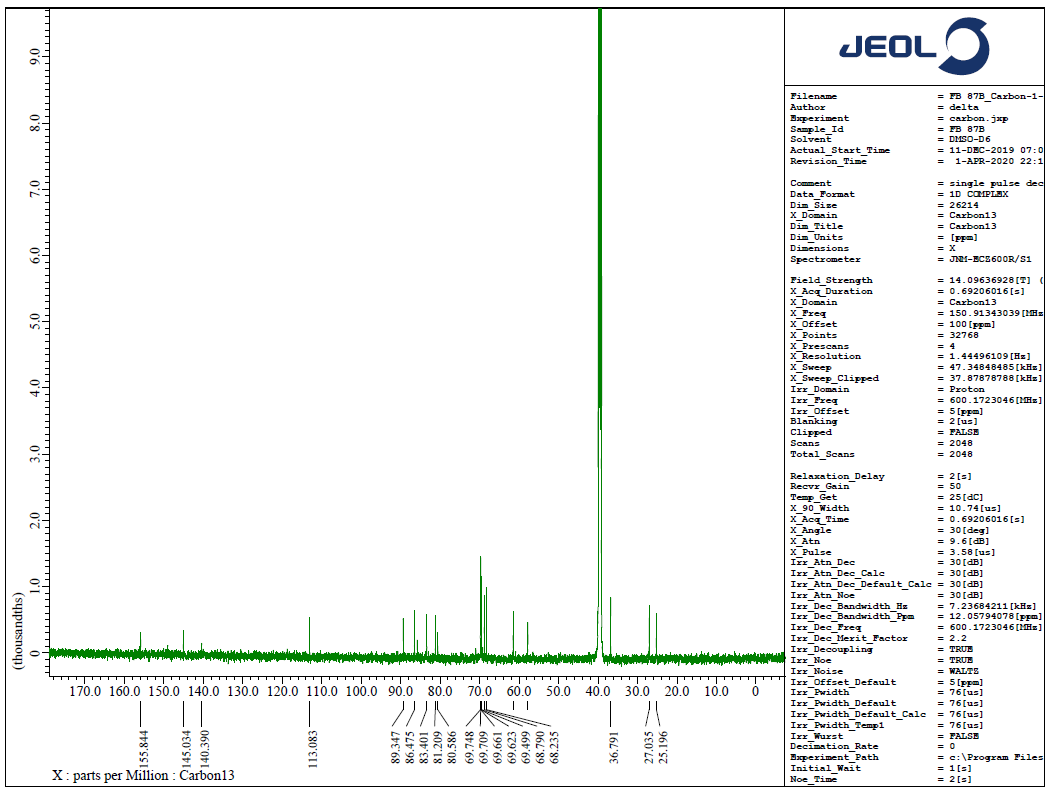


**11c. ^1^H NMR spectrum**

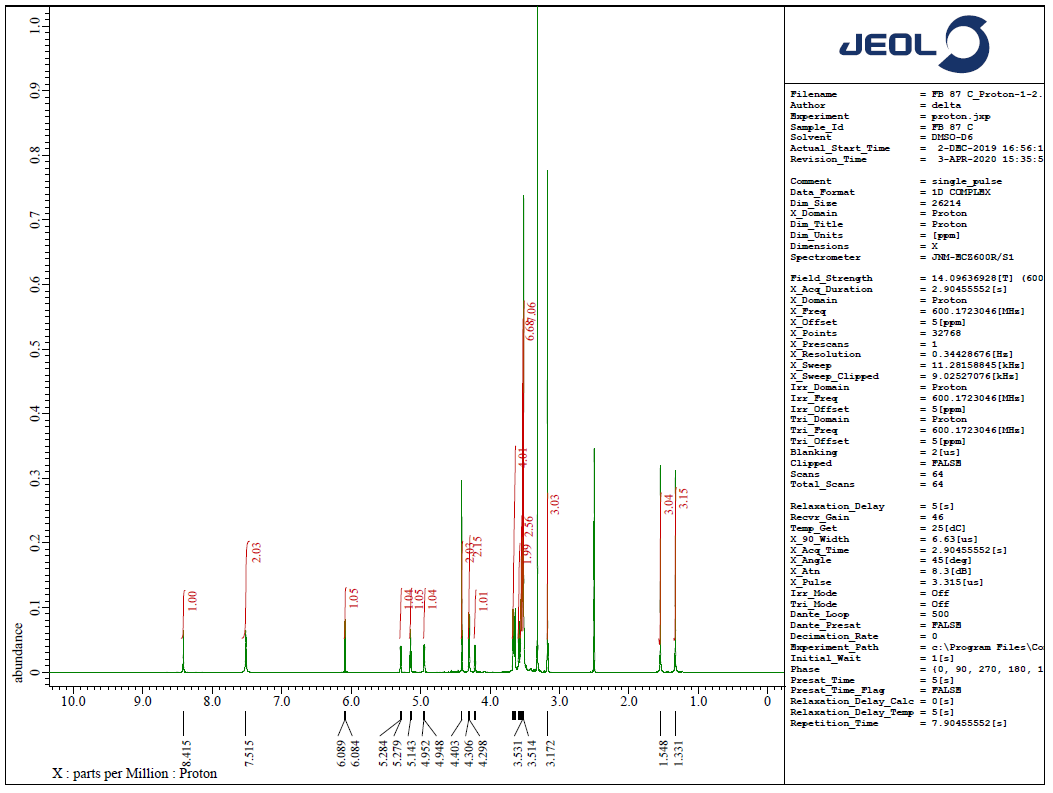


**11c. ^13^C NMR spectrum**


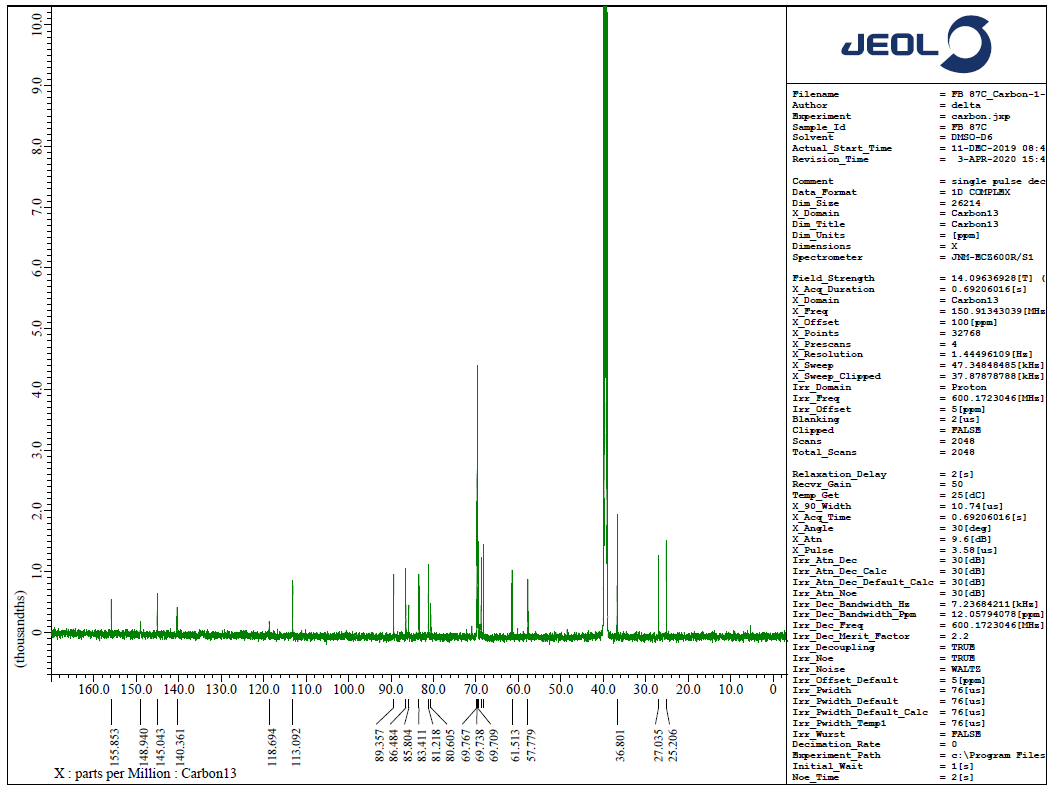


**12c. ^1^H NMR spectrum**

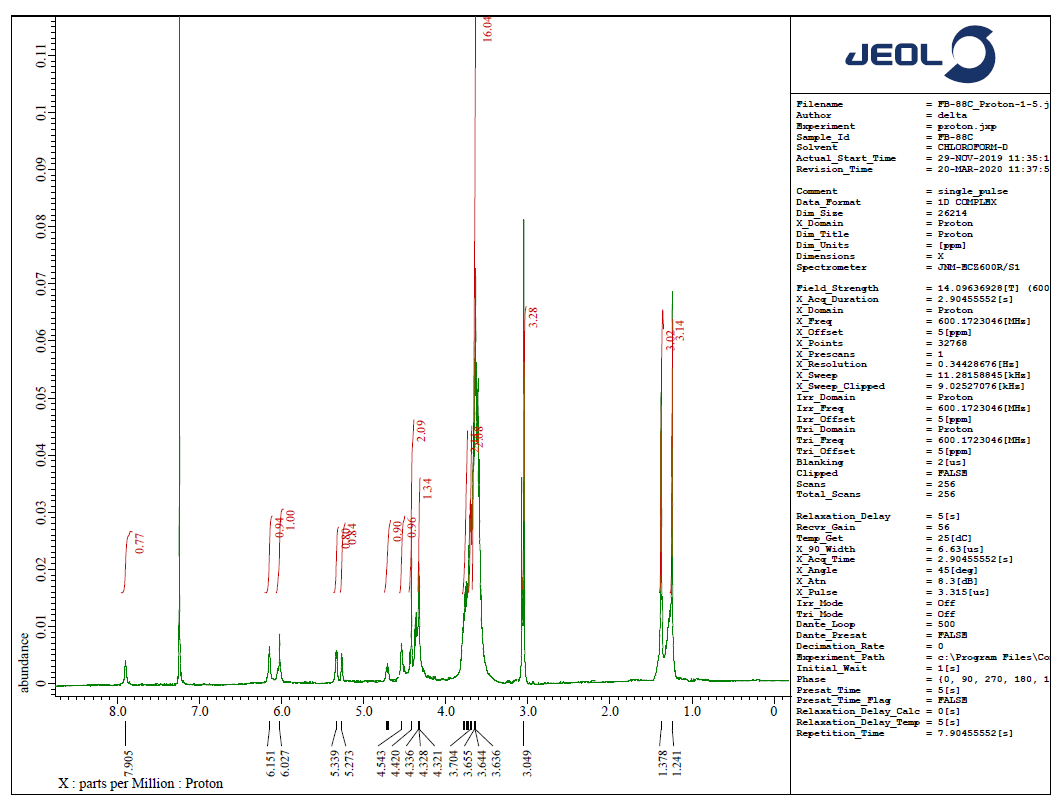


**12d. ^1^H NMR spectrum**

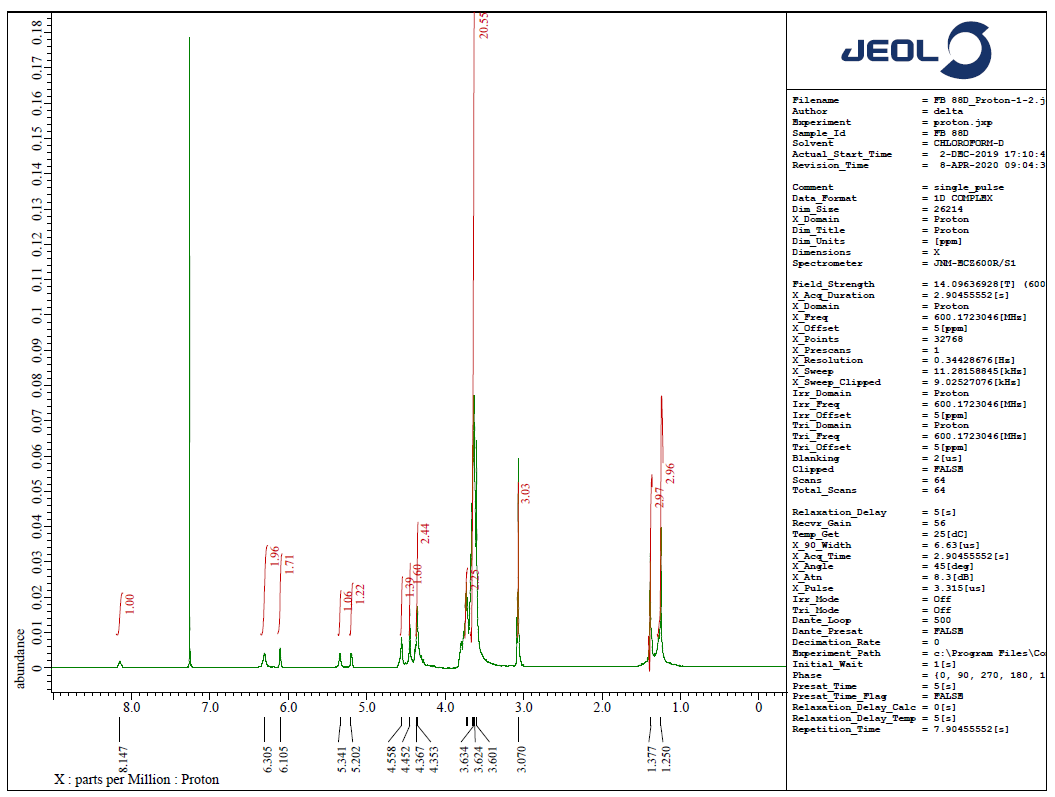


**A_1_ A_2A_ and A_3_ AR. Proteins alignment (top view)**


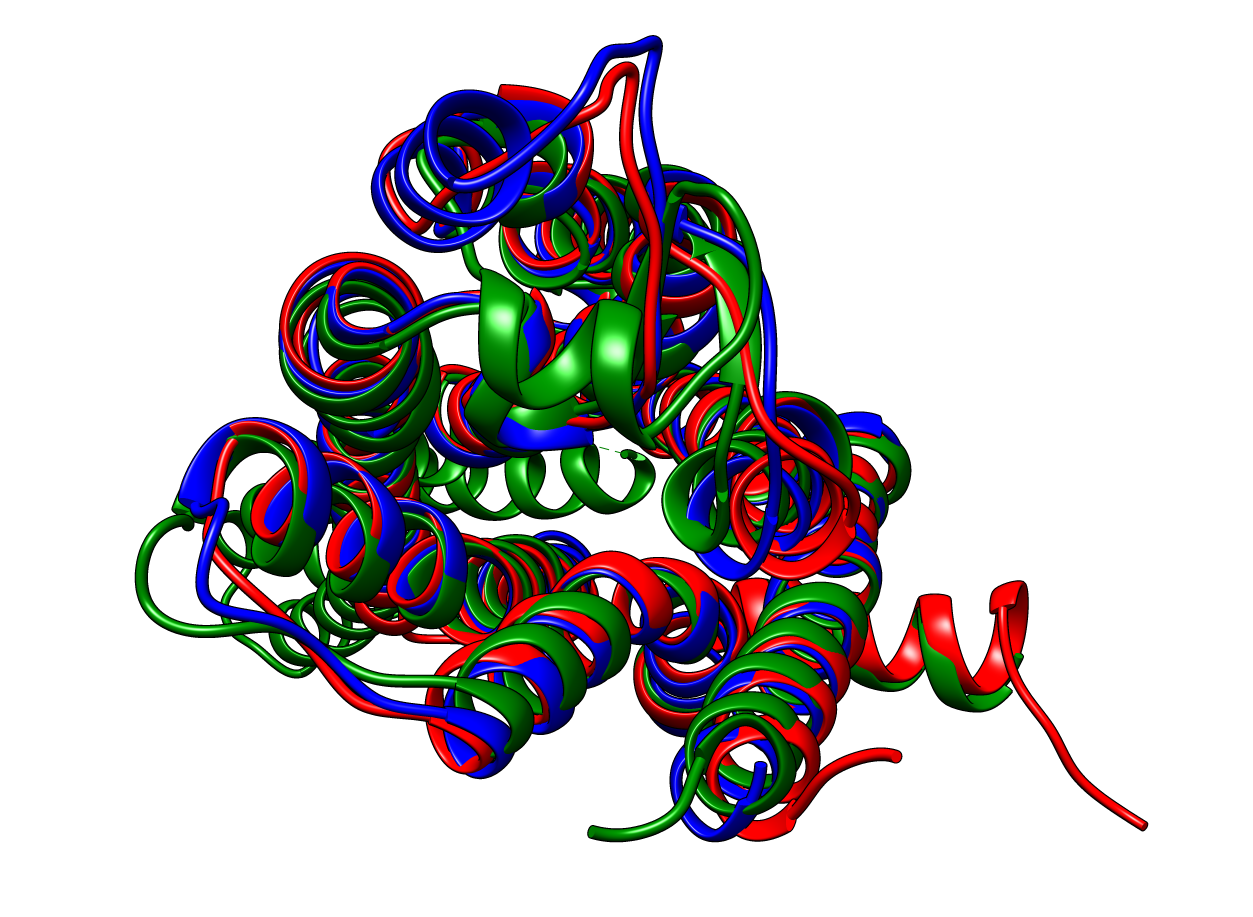


**Legend**. The A_1_ receptor is shown in blue, the A_2A_ receptor in green and the A_3_ receptor in red.

**Protein alignment (side view)**


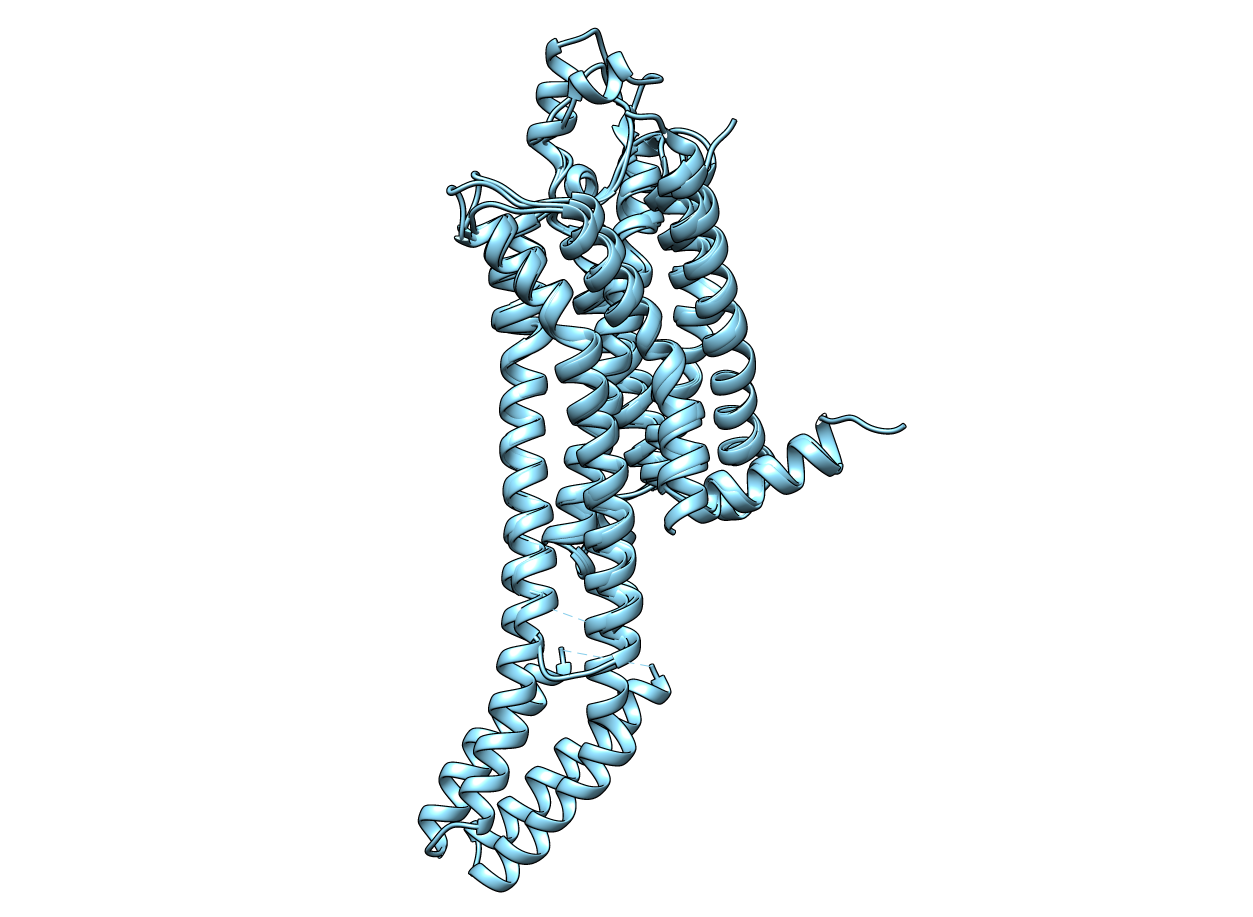


| **Compound (top view)** | **A1 Receptor** | **A2 Receptor** | **A3 Receptor** |
| --- | --- | --- | --- |
| **Hexynyladenosine (2)** | 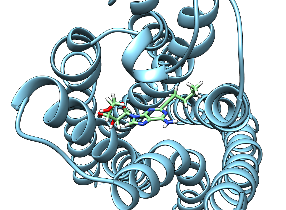 | 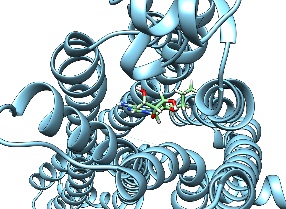 | 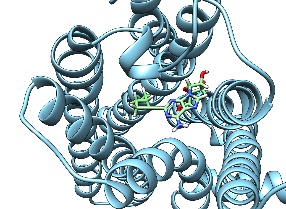 |
| **Phenylethynyladenosine (3)** | 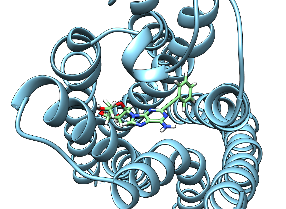 | 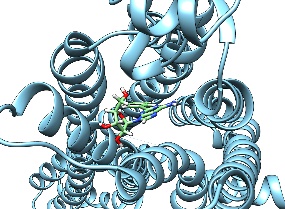 | 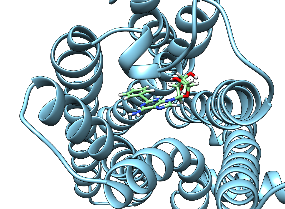 |
| **10c** | 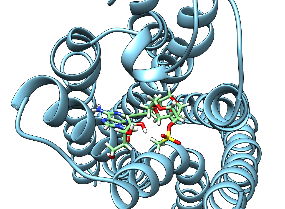 | 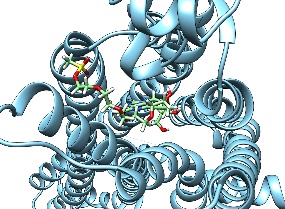 | 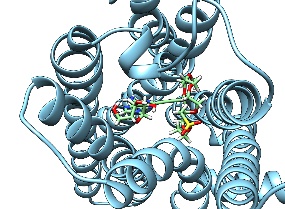 |
| **11c** | 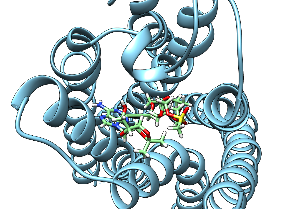 | 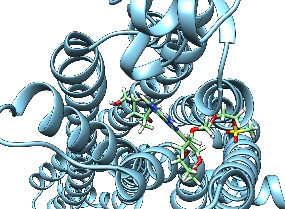 | 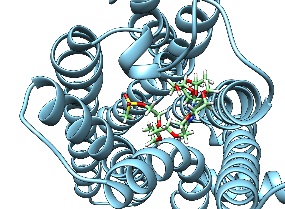 |
| **12c** | 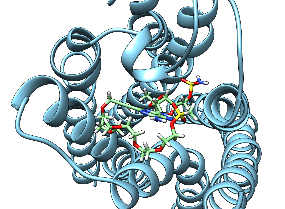 | 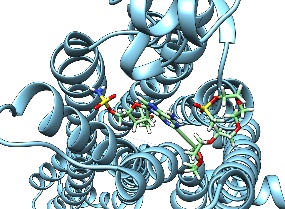 | 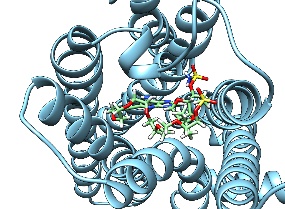 |

| **Compound (side view)** | **A1 Receptor** | **A2 Receptor** | **A3 Receptor** |
| --- | --- | --- | --- |
| **Hexynyladenosine (2)** | 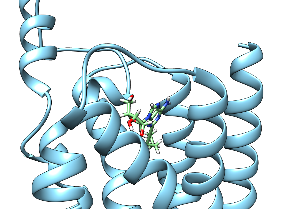 | 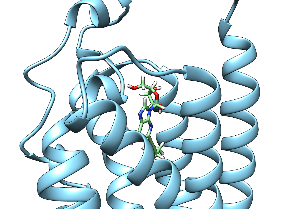 | 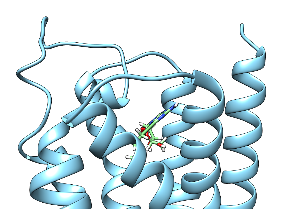 |
| **Phenylethynyladenosine (3)** | 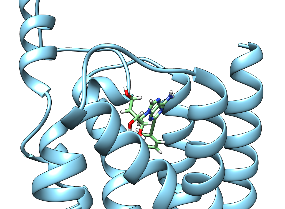 | 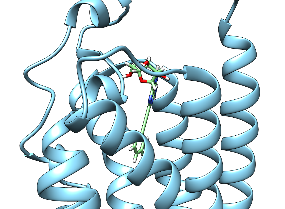 | 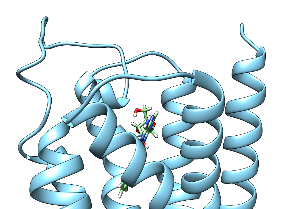 |
| **10c** | 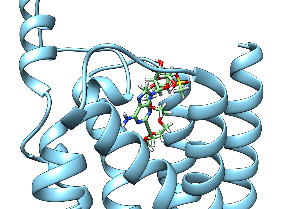 | 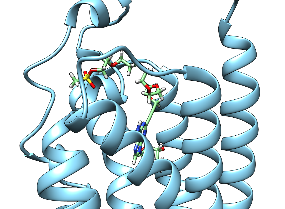 | 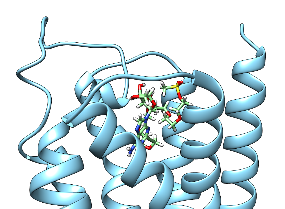 |
| **11c** | 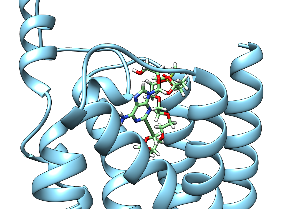 | 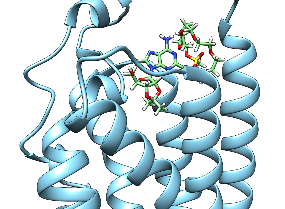 | 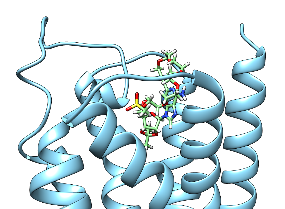 |
| **12c** | 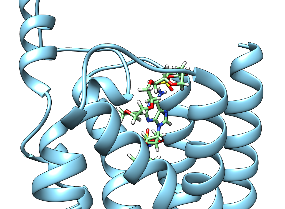 | 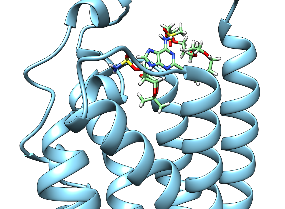 | 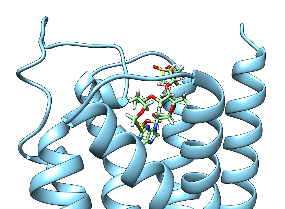 |

**Solubility determination of Poly-ethylene glycol (PEG)adenosine conjugates.**

**Table 1**. HPLC analysis of PEG-AND conjugates in deuterium-depleted water (DDW) and phosphate buffer solution.^a^

| **PEG-AND conjugates** | **Wavelength (nm)** | **Retention time (min)** |
| --- | --- | --- |
| **10b** | 230 | 11.074(DDW)  11.081 (PBS) |
| **11b** | 230 | 11.208 (DDW)  11.205 (PBS) |

*^a^*Column: RP-18 (5µm) 150 × 4.6 mm; mobile phase (v/v) gradient elution: deionized water/acetonitrile (0.1 % Formic acid); flow rate: 1.0 mL/min; Run time = 13.6 min.

**Plots for solubility determination.**

A linear concentration ranging from 5.0–1000.0 µg/ml was employed in the experiments. Good linearity (r2 > 0.9995) was achieved in PEG-AND conjugates **10b**, **11b** and adenosine. The aqueous solubility of PEG-AND conjugate **10b** was calculated to be 1.33 mg/mL in DDW and 1.22 mg/mL in PBS.

The aqueous solubility of PEG-AND conjugate **11b** was calculated to be 1.16 mg/mL in DDW and 1.18 mg/mL in PBS.
