# Supplementary material for: C2‐linked alkynyl poly‐ethylene glycol(PEG) adenosine conjugates as water‐soluble adenosine receptor agonists
Source: Chem Biol Drug Des. 2022 Aug 22;101(2):340–9. doi: 10.1111/cbdd.14128 (PMC10087458; doi:10.1111/cbdd.14128)
Supplement: Supplementary file 2 — Data S1 [file CBDD-101-340-s001.docx]

**Experimental**

**General chemistry methods**

^1^H- and ^13^C-Nuclear Magnetic Resonance (NMR) spectroscopy analyses were carried out using a JEOL JNM-ECZR 600 MHz (equipped with a ROYAL probe) or Bruker Avance 400 MHz NMR spectrometers. Solvent signals for hydrogen and carbon NMR were used as the internal reference. Chemical shifts (*δ*_H_) are quoted in parts per million and are relative to the solvents residual peaks in the ^1^H and ^13^C NMR spectra: CDCl_3_ (7.26 and 77.0 ppm), MeOD-*d*_4_ (3.31 and 49.1 ppm) and DMSO-*d*_6_ (2.50 and 39.52 ppm). Coupling constants (J) are given in Hertz (Hz) and the signal multiplicity is described as singlet (s), doublet (d), doublet of doublets (dd), triplet of doublets (td), triplet (t), quartet (q) and multiplet (m).

Chemicals were purchased from Acros Organic, Alfa Aesar, Fisher Scientific, Sigma Aldrich and VWR. The deuterated solvents (CDCl_3_, DMSO‑*d*_6_ and MeOD-*d*_4_) used for NMR spectroscopy experiments were purchased from Cambridge Isotope Laboratories Inc.

All moisture sensitive materials were handled under nitrogen atmosphere via use of standard Schlenk line techniques, glassware was flame-dried under vacuum and anhydrous solvents were distilled before use.

Thin Layer Chromatography (TLC) was performed using aluminium backed 20 × 20 cm silica gel 60 F_254_, which were purchased from Merck for viewing colourless spots under 254 nm wavelength ultraviolet light.

Purifications of the intermediates and final products were conducted using an Interchim Puriflash 4100 automatic purification machine. The crude compounds were injected into the system at a rate of 5 ml/min at 100 bar, using a 15 µg silica, high-capacity column. Alternatively, flash column chromatography purifications were conducted in a glass column using irregular, 60 Å pore size silica gel, 63-200 μm, 70-230 mesh.

LC-MS analysis was conducted on a Thermo Fisher – Agilent 6100 series Quadrupole LC-MS system with a G4220A 1290 binary pump/DAD. The column used was an Agilent Zorbax SB-C18 2.1 x 50 mm 1.8 micron (400 bar). High pressure was typically starting at about 500 psi at 5% acetonitrile (ACN) within a 13.60 min run. The flow was 1 mL/min. Solution A: H_2_O 0.1% formic acid. Solution B: ACN 0.1% formic acid. The method was as follows: Solution B at 5% for 8 min, then a gradient up to 100% B for 1 min, then hold at 100% for 2 min, then back to 5% ACN over 0.5 min. Then hold at 5% until 13.60 min.

General procedure A for the preparation of **10a-d.** Each of the mesylated alkynyl-PEG_3-6_ derivatives **8a-d** (3.0 equiv.) was added dropwise via syringe pump over 0.5 h to separated solutions of 2‑iodoadenosine **9** (250 mg, 0.64 mmol, 1.0 equiv.), dichlorobis(triphenylphospine)palladium (II) (0.05 equiv.), and copper iodide (0.076 equiv.) in dry CH_3_CN/Et_3_N (1:1 *v/v*, 10 mL). The reaction was stirred 4 h at 23 °C, then concentrated *in vacuo*. Purification by flash chromatography (0–10% EtOAc-MeOH) afforded the title compounds.

General procedure B for the preparation of **11a-d.** To a solution of **10a-d** (1.0 equiv.) in acetone (10 mL) was added 2,2-dimethoxypropane (DMP) (4.9 equiv.) and *p*-toluenesulfonic acid (1.09 equiv.). The reaction was stirred at 23 °C for 16 h. To the resulting suspension was added solid NaHCO_3_ to quench the reaction and then the acetone was removed *in vacuo*. The residue was partitioned between EtOAc (20 mL) and saturated aqueous NaHCO_3_. The aqueous layer was further extracted with EtOAc (3 × 20 mL). The combined extracts were washed with saturated aqueous NaCl (50 mL), dried (MgSO_4_), and concentrated *in vacuo*. Purification by flash chromatography (SiO_2_, EtOAc-MeOH 0–20%) afforded the title products.

General procedure C for the preparation of **12a-d.** To a solution of **11a-d** (1 equiv.) in DME (10 mL) was added sodium hydride (60% dispersion in mineral oil, 2.75 equiv.) at 0 °C. After 0.5 h, solid sulfamoyl chloride (2 equiv.) was added in one portion and the reaction was stirred for 1 h at room temperature. The reaction mixture was quenched with MeOH (5 mL), filtered through Celite and purified by flash chromatography (SiO_2_, EtOAc-MeOH 0–20%) to afford the title compounds.

**2-(2-(2-((3-(6-amino-9-((2*S*,3*S*,4*R*,5*S*)-3,4-dihydroxy-5-(hydroxymethyl) tetrahydrofuran-2-yl)-9H-purin-2-yl)prop-2-yn-1-yl)oxy)ethoxy)ethoxy)ethyl methanesulfonate (10a).** 2-(2-(2-(prop-2-yn-1-yloxy)ethoxy)ethoxy)ethylmethanesulfonate **8a** was reacted with 2‑iodoadensine **9** according to the general procedure A to afford **10a** (214 mg, 79%) as a dark brown oil. *R_f_* = 0.34 (EtOAc-MeOH 8:2 *v/v*); MS *m/z* 532 (M^+^); ^1^H-NMR (600 MHz, DMSO-*d*_6_) *δ*_H_ 8.43 (s, 1H, H8), 7.50 (s, 2H, NH_2_), 5.86 (d, J = 6.2 Hz, 1H, H-1′), 5.45 (d, J = 6.2 Hz, 1H), 5.18-5.16 (m, 2H), 4.53 (q, J = 5.6 Hz, 1H, H-2′), 4.40 (s, 2H), 4.32-4.30 (m, 1H, H-3′), 4.14-4.11 (m, 1H, H-4′), 3.95 (q, J = 3.6 Hz, 1H, H-5′), 3.69-3.67 (m, 2H), 3.66-3.64 (m, 2H), 3.60-3.55 (m, 8H), 3.18 (s, 3H), 3.11-3.08 (m, 2H); ^13^C-NMR (151 MHz, DMSO-*d*_6_) *δ*_C_ 155.8, 144.9, 140.5, 87.4, 85.7, 80.5, 73.6, 70.9, 70.4, 69.8, 69.5, 69.3, 68.8, 61.4, 57.7, 36.8; HRMS: found 531.1637calculated for C_20_H_29_N_5_O_10_S 531.1635.

**15-(6-amino-9-((2*R*,3*R*,4*S*,5*R*)-3,4-dihydroxy-5-(hydroxymethyl)tetrahydrofuran-2-yl)-9H-purin-2-yl)-3,6,9,12-tetraoxapentadec-14-yn-1-yl methanesulfonate (10b).** 3,6,9,12-tetraoxapentadec-14-yn-1-yl methanesulfonate **8b** was reacted with 2-iodoadenosine **9** according to the general procedure A to afford **10b** (200 mg, 55%) as a brown oil. *R_f_ =* 0.29 (EtOAc-MeOH 8:2 *v/v* ); MS *m/z* 576 (M^+^); ^1^H-NMR (600 MHz, DMSO-*d*_6_) *δ*_H_ 8.43 (s, 1H), 7.50 (s, 2H, NH_2_), 5.86 (d, J = 5.9 Hz, 1H, H-1′), 5.46 (d, J = 6.2 Hz, 1H), 5.18-5.17 (m, 2H), 4.53 (q, J = 5.5 Hz, 1H, H-2′), 4.40 (s, 2H), 4.31-4.29 (m, 1H, H-3′), 4.13 (q, J = 4.1 Hz, 1H, H-4′), 3.95 (q, J = 3.6 Hz, 1H, H-5′), 3.67-3.63 (m, 4H), 3.59-3.58 (m, 2H), 3.57-3.55 (m, 4H), 3.54 (d, J = 3.3 Hz, 10H), 3.17 (s, 3H), 3.11-3.09 (m, 2H); ^13^C-NMR (151 MHz, DMSO-*d*_6_) *δ*_C_ 155.8, 144.9, 140.6, 87.5, 85.7, 80.5, 73.6, 70.9, 70.4, 69.7, 69.6, 69.5, 69.3, 68.8, 68.2, 61.4, 57.7, 36.8; HRMS: found 575.1892 calculated for C_22_H_33_N_5_O_11_S 575.1897.

**18-(6-amino-9-((2*R*,3*R*,4*S*,5*R*)-3,4-dihydroxy-5-(hydroxymethyl)tetrahydrofuran-2-yl)-9H-purin-2-yl)-3,6,9,12,15-pentaoxaoctadec-17-yn-1-yl methanesulfonate (10c). 8c** was reacted with 2-iodoadenosine **9** according to the general procedure A to afford **10c** (250 mg, 88%) as a brown oil. *R_f_* = 0.22 (EtOAc-MeOH 8:2 *v/v* ); MS *m/z* 620 (M^+^); ^1^H-NMR (600 MHz, DMSO-*d*_6_) *δ*_H_ 8.43 (s, 1H, H-8), 7.50 (s, 2H, NH_2_), 5.86 (d, J = 5.9 Hz, 1H, H-1′), 5.45 (d, J = 6.2 Hz, 1H), 5.17 (t, J = 5.7 Hz, 2H), 4.53 (q, J = 5.6 Hz, 1H, H-2′), 4.40 (s, 2H), 4.31-4.29 (m, 1H, H-3′), 4.14-4.12 (m, 1H, H-4′), 3.95 (q, J = 3.6 Hz, 1H, H-5′), 3.67-3.63 (m, 2H), 3.59-3.57 (m, 2H), 3.55-3.54 (m, 4H), 3.53-3.51 (m, 12H), 3.17 (s, 3H), 3.11-3.09 (m, 2H); ^13^C-NMR (151 MHz, DMSO-*d*_6_) *δ*_C_ 155.8, 144.9, 140.6, 118.9, 87.4, 85.7, 80.5, 73.6, 70.9, 70.4, 69.7, 69.6, 69.5, 69.3, 68.8, 68.2, 61.4, 57.7, 36.8; HRMS: found 619.2157 calculated for C_24_H_37_N_5_O_12_S 619.2159.

**21-(6-amino-9-((2*R*,3*R*,4*S*,5*R*)-3,4-dihydroxy-5-(hydroxymethyl)tetrahydrofuran-2-yl)-9H-purin-2-yl)-3,6,9,12,15,18-hexaoxahenicos-20-yn-1-yl methanesulfonate (10d). 8d** was reacted with 2-iodoadenosine **9** according to the general procedure A to afford **10d** (340 mg, 80%) as a brown oil. *R_f_* = 0.22 (EtOAc-MeOH 8:2 *v/v* ); MS *m/z* 664 (M^+^1); ^1^H-NMR (600 MHz, CDCl_3_) *δ*_H_ 8.03 (s, 1H, H-8), 6.89 (s, 1H), 5.84 (d, J = 7.1 Hz, 1H, H-1′), 5.61 (s, 1H), 5.12 (s, 1H), 4.38-4.36 (m, 1H), 4.34 (d, J = 4.5 Hz, 1H), 4.30-4.28 (m, 2H), 3.90 (d, J = 12.4 Hz, 1H), 3.76-3.73 (m, 2H), 3.72-3.71 (m, 2H), 3.67-3.62 (m, 20H), 3.08 (s, 3H); ^13^C-NMR (151 MHz, CDCl_3_) *δ*_C_ 156.3, 148.0, 144.5, 142.2, 90.6, 87.1, 82.4, 73.3, 72.9, 72.1, 70.7, 70.6, 70.3, 69.5, 69.1, 63.6, 58.7, 37.9; HRMS: found 663.2420 calculated for C_26_H_41_N_5_O_13_S 663.2422.

**2-(2-(2-((3-(6-amino-9-((3a*S*,4*S*,6*S*,6a*S*)-6-(hydroxymethyl)-2,2-dimethyltetrahydrofuro[3,4-d][1,3]dioxol-4-yl)-9H-purin-2-yl)prop-2-yn-1-yl)oxy)ethoxy)ethoxy)ethyl methanesulfonate (11a).** Acetonide protection of **10a** was accomplished according to the general procedure B to afford **11a** (140 mg, 82%) as a yellow oil. MS *m/z* 572 (M^+^); ^1^H-NMR (400 MHz, CD_3_OD) *δ*_H_ 8.36 (s, 1H, H-8), 6.14 (d, J = 3.57 Hz, 1H, H-1′), 5.25 (dd, *J* = 5.95, 3.43 Hz, 1H), 5.04 (dd, *J* = 6.09, 2.35 Hz, 1H), 4.45 (s, 2H), 4.37-4.35 (m, 3H, CH_2_), 3.82-3.67 (m, 15H), 3.67 (s, 3H), 3.12 (s, 3H), 1.62 (s, 3H), 1.39 (s, 3H). ^13^C-NMR (100 MHz, CD_3_OD): *δ*_C_ 115.3, 92.7, 88.2, 86.1, 85.3, 82.9, 71.6, 71.4, 71.0, 70.5, 70.1, 63.6, 59.4, 37.6, 27.6, 25.6; HRMS: found 571.1945 calculated for C_23_H_33_N_5_O_10_S 571.1948.

**15-(6-amino-9-((3*aR*,4*R*,6*R*,6a*R*)-6-(hydroxymethyl)-2,2-dimethyltetrahydrofuro[3,4-d][1,3]dioxol-4-yl)-9H-purin-2-yl)-3,6,9,12-tetraoxapentadec-14-yn-1-yl methanesulfonate (11b).** Acetonide protection of **10b** was accomplished according to the general procedure B to afford **11b** (180 mg, 41%) as a dark yellow oil. *R_f_* = 0.32 (EtOAc-MeOH: 8:2 *v/v*); MS *m/z* 616 (M^+^); ^1^H-NMR (600 MHz, DMSO-*d*_6_) *δ*_H_ 8.42 (s, 1H, H8), 7.52 (s, 2H, NH_2_), 6.09 (d, J = 3.0 Hz, 1H, H-1′), 5.29 (dd, J = 6.2, 3.0 Hz, 1H), 5.14 (t, J = 5.5 Hz, 1H), 4.94 (dd, J = 6.2, 2.6 Hz, 1H, H-2′), 4.40 (s, 2H), 4.31-4.29 (m, 1H, H-3′), 4.24 (td, J = 4.7, 2.7 Hz, 1H, H-4′), 3.68-3.66 (m, 3H), 3.65-3.63 (m, 3H), 3.60-3.58 (m, 2H), 3.56-3.55 (m, 2H), 3.54-3.54 (m, 12H), 3.17 (s, 3H), 1.55 (s, 3H), 1.33 (s, 3H); ^13^C-NMR (151 MHz, DMSO-*d*_6_) *δ*_C_ 155.8, 145.0, 140.4, 113.1, 89.3, 86.5, 83.4, 81.2, 80.6, 69.7, 69.7, 69.7, 69.6, 69.5, 68.8, 68.2, 61.5, 57.8, 36.8, 27.0, 25.2; HRMS: found 615.2208 calculated for C_25_H_37_N_5_O_11_S 615.2210.

**18-(6-amino-9-((3a*R*,4*R*,6*R*,6a*R*)-6-(hydroxymethyl)-2,2-dimethyltetrahydrofuro[3,4-d][1,3]dioxol-4-yl)-9H-purin-2-yl)-3,6,9,12,15-pentaoxaoctadec-17-yn-1-yl methanesulfonate (11c).** Acetonide protection of **10c** was accomplished according to the general procedure B to afford **11c** (60 mg, 30%) as a yellow oil. *R_f_* = 0.35 (EtOAc-MeOH 8:2 *v/v*); MS *m/z* 660 (M^+^); ^1^H-NMR (600 MHz, DMSO-*d*_6_) *δ*_H_ 8.41 (s, 1H, H8), 7.51 (s, 2H, NH_2_), 6.09 (d, J = 3.1 Hz, 1H, H-1′), 5.29 (q, J = 3.1 Hz, 1H), 5.14 (t, J = 5.5 Hz, 1H), 4.94 (q, J = 3.0 Hz, 1H, H-2′), 4.40 (s, 2H), 4.31-4.29 (m, 1H, H-3′), 4.22 (td, J = 4.6, 2.7 Hz, 1H, H-4′), 3.67-3.63 (m, 4H), 3.59-3.57 (m, 2H), 3.55-3.55 (m, 2H), 3.53 (s, 6H), 3.51 (s, 6H), 3.17 (s, 3H), 1.55 (s, 3H), 1.33 (s, 3H). ^13^C-NMR (151 MHz, DMSO-*d*_6_) *δ*_C_ 155.9, 148.9, 145.0, 140.4, 118.7, 113.1, 89.4, 86.5, 85.8, 83.4, 81.2, 80.6, 69.8, 69.7, 69.7, 69.7, 69.6, 69.5, 68.8, 68.2, 61.5, 57.8, 36.8, 27.0, 25.2; HRMS: found 659.2472 calculated for C_27_H_41_N_5_O_12_S 659.2472.

**2-(2-(2-((3-(6-amino-9-((3a*S*,4*S*,6*S*,6a*S*)-6-(hydroxymethyl)-2,2-dimethyltetrahydrofuro[3,4-d][1,3]dioxol-4-yl)-9H-purin-2-yl)prop-2-yn-1-yl)oxy)ethoxy)ethoxy)ethyl methanesulfonate-5′-O-(sulfamoyl)adenosine (12a).** The title compound **12a** was obtained (55 mg, 0.085 mmol, 35%) according to general procedure C starting from **11a** (0.14 g, 0.245 mmol). (EtOAc-MeOH 0–20%); *R_f_* = 0.45 (EtOAc : MeOH : 8:2 *v/v* ); MS *m/z* 651 (M^+^); ^1^H-NMR (400 MHz, CD_3_OD) *δ*_H_ 8.30 (s, 1H, H-8), 6.24 (d, *J* = 2.48 Hz, 1H, H-1′), 5.38 (dd, *J* = 6.19, 2.55 Hz, 1H), 5.14 (dd, *J* = 6.35, 2.85 Hz, 1H), 4.52 (m, 1H, H-2′), 4.46 (s, 2H), 4.38-4.35 (m, 2H, CH_2_), 4.31-4.25 (m,1H), 3.81-3.76 (m, 5H), 3.72-3.67 (m, 7H), 3.12 (s, 3H), 1.61 (s, 3H, CH_3_), 1.40 (s, 3H, CH_3_). ^13^C-NMR (100 MHz, CD_3_OD): *δ*_C_ 115.7, 91.8, 85.9, 85.6, 83.1, 82.7, 71.6, 71.4, 71.0, 70.5, 70.1, 70.0, 59.4, 37.6, 30.7, 27.5, 25.6; HRMS: found 650.1673 calculated for C_23_H_34_N_6_O_12_S_2_ 650.1676.

**Microbiology and cytotoxicity experiments**

Slow growing *Mycobacterium bovis* BCG (ATCC35734), and fast and *Mycobacterium aurum* (ATCC 23366) were used for determining the anti-mycobacterial activity of the chemical compounds studied in this work. The MIC determination using solid agar method (Spot Culture Growth Inhibition assay) and the resazurin cytotoxicity assay using RAW264.7 macrophages procedures were previously reported.^31,39,40,41^

**RAW264.7 murine macrophage culture for intracellular cAMP measurements**

RAW 264.7 mouse macrophage cell line was a kind gift from Dr. Gareth Williams at the School of Pharmacy, University College London. Cells were thawed quickly in a water bath and resuspended in DMEM (Lonza, UK, LZBE12-604F), supplemented with 10% FBS (Sigma, UK, F9665-500ML), without antibiotics, and incubated at 5% CO_2_ and 37ºC. After incubation with the cells, the cell free supernatant was tested for mycoplasma using the MycoAlert Mycoplasma Detection Kit (Lonza, UK, LT07-118) and MycoAlert Assay Control set (Lonza, UK, LT07-518) as positive and negative controls to confirm cells were mycoplasma free, following the manufacturer instructions. Media was replenished every 3 days.

**Intracellular cAMP measurements**

RAW 264.7 macrophages were seeded in a 12-well plate at a density of 0.5 x 10^6 per well, and serum was reduced to 5 % to prevent growth limit. After 24-hr cells were pre-treated with 4-(3-butoxy-4-methoxybenzyl)-2-imidazolidinone 100 nM to inhibit Phosphodiesterase enzyme (PDE) and left to incubate for 10 minutes, before being treated with either forskolin (1 μM), adenosine (1 μM), **10b** (1 μM) or **11b** (1 μM) for an additional 10 minutes. Post incubation, cells were washed with sterile PBS, lysed with 0.1 M HCl and cytoplasmic cell content was used for further analysis. cAMP was measured using a competitive cAMP ELISA kit (ADI-900-163-Enzo life sciences, Farmingdale, NY, USA) following the manufacturer’s instructions. Absorbance was measured at 405 nm using Promega GloMax Discovery system Microplate Reader. Measurements were performed in triplicates.

**Solubility determination of Poly-ethylene glycol (PEG)adenosine conjugates:**

The equilibrium solubility of PEG-ADN conjugates was determined in 0.1 M phosphate buffer solution (pH = 6.8) and in DDW (pH = 7). An excess amount of the PEG-ADN conjugates was added into a 20 mL glass vial containing either phosphate buffer solution or DDW. The samples were placed into a shaking water bath at 150 rpm at 37ᵒC for 24h. The supernatant was withdrawn from the suspension using a micro centaur MSB010.CX2.5 centrifuge (MSE Ltd., London, UK) at 1.3 × 10,000 rpm for 1 min.

The supernatant was decanted and then diluted with ethanol to prevent crystallisation (dilution factor: 2:1). The concentrations of PEG-ADN conjugates were determined using high-pressure liquid chromatography (HPLC) analysis with ultra-violet (UV) spectrometric detection (230 nm). All experiments were conducted in triplicates. The injection volume was 20 μL. The HPLC instrument consisted of an Agilent 1100 Autosampler, G1315B Diode Array Detector, G1311A Quaternary pump, and the column used was a Kinetex 5 μm C18 100 Å LC 150 × 4.6 mm. Solution A: H_2_O with 0.1% formic acid. Solution B: ACN with 0.1% formic acid. The flow rate was 1 mL/min. Solution B increased from 5% to 100% over a 8 min period and reverted to 5% until 13.60 min.

**Molecular modelling**

The ligands and the native agonist adenosine were drawn in ChemDraw and converted to a 3D format in Chem3D (ChemOffice 19.1, Perkin Elmer). The structures were energy minimised in Chem3D using the MM2 and MMFF94 protocols using the default parameters. The molecules were saved in mol2 format and converted to pdb and pdbqt file formats using Openbabel version 2.3.1.^47^

The adenosine receptor structures with the native agonist adenosine bound were downloaded from the protein databank for the A_1_ and A_2A_ receptors, e.g., PDB ID 7LD4^42^ and 2YDO^43^ for A_1_ and A_2A_ ARs, respectively. There was no crystal structure for the A_2B_ and A_3_ receptor, so the Alphafold^44^ structures for the human proteins were downloaded from the Uniprot website (https://www.uniprot.org, accessed 20-05-22). For the A_2B_ and A3 receptor proteins, the proteins were overlaid with the A_2A_ receptor structure containing the adenosine ligand. The A_2B_ (residues 1-312) and A3 (residues 1-303) receptors containing the adenosine in the binding pocket were energy minimised using AMBER20^46^ (FF14SB force field for the protein, GAFF parameters for adenosine, 10 Å TIP3P truncated octahedral water box, 0.15 M potassium chloride) and subjected to a short 80 ps restrained molecular dynamics simulation to optimise the protein-ligand interactions. All proteins were overlaid using UCSF Chimera 1.16^48^ and solvent, counterions and ligands were removed. The structures were converted to the pdbqt file format using Autodock Tools 1.5.6^49^ and a grid (25 × 25 × 37 Å) was defined around the adenosine binding site of the aligned protein structures using Autodock Tools. The ligands were docked to the protein using Autodock Vina 1.2.3,^45^ with default parameters and an exhaustiveness of 24. The ligand conformations with the lowest estimated binding energy were saved and visualised using Chimera.
