# Supplementary material for: C2‐linked alkynyl poly‐ethylene glycol(PEG) adenosine conjugates as water‐soluble adenosine receptor agonists
Source: Chem Biol Drug Des. 2022 Aug 22;101(2):340–9. doi: 10.1111/cbdd.14128 (PMC10087458; doi:10.1111/cbdd.14128)
Supplement: Supplementary file 3 — Figure S1 [file CBDD-101-340-s003.docx]

**Figure 5a-h**

**
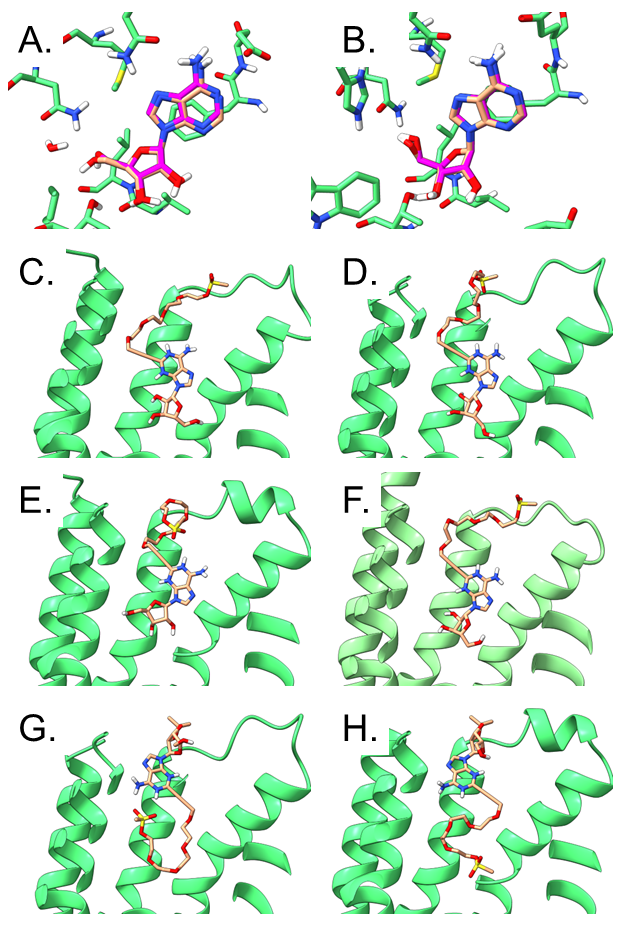
**

**Figure 5a-f.** **a.** Docked conformations of adenosine (tan stick representation) in complex with the A_1_ receptor (green ribbon representation) overlayed with the experimental structure (magenta stick representation); **b.** Docked conformations of adenosine in complex with the A_2A_ receptor (tan) overlayed with the experimental structure (magenta); **c-f.** Docked conformation of compound **11b** bound to the A_1_ (c), A_2A_ (d) A_2B_ (e) and A_3_ (f) receptors; **g-h.** Docked conformation of compound **10b** bound to the A_2A_ (g) and A_2B_ (h) receptors. Structures **c**-**h** are rotated 180° relative to **a**-**b** so that a slice-through representation of the protein could be used to produce comparable images.
